# The Effect of Repetitive Transcranial Magnetic Stimulation of the Dorsolateral Prefrontal Cortex on the Amyotrophic Lateral Sclerosis Patients With Cognitive Impairment: A Double‐Blinded, Randomized, and Sham Control Trial

**DOI:** 10.1111/cns.70316

**Published:** 2025-03-18

**Authors:** Wensi Zheng, Xiaojie Zhang, Jingjiong Chen, Xinghua Luan, Jijun Wang, Liren Zhang, Kun Liu, Yuwu Zhao, Zhouwei Xu

**Affiliations:** ^1^ Shanghai Key Laboratory of Psychotic Disorders, Shanghai Mental Health Center Shanghai Jiao Tong University School of Medicine Shanghai China; ^2^ Department of Neurology Shanghai Sixth People's Hospital Affiliated to Shanghai Jiao Tong University School of Medicine Shanghai China; ^3^ Shanghai Neurological Rare Disease Biobank and Precision Diagnostic Technical Service Platform Shanghai China; ^4^ Department of Rehabilitation Medicine Shanghai Sixth People's Hospital Affiliated to Shanghai Jiao Tong University School of Medicine Shanghai China

**Keywords:** amyotrophic lateral sclerosis, caregiver's burden, cognitive impairment, prognosis, transcranial magnetic stimulation

## Abstract

**Background:**

Amyotrophic lateral sclerosis (ALS) is a neurodegenerative disease. A large number of ALS patients have cognitive impairment. In this double‐blinded, randomized, and sham‐controlled study, we aimed to investigate the effect of repetitive transcranial magnetic stimulation (rTMS) on ALS patients with cognitive impairment.

**Methods:**

A total of 90 ALS patients with cognitive impairment were recruited from two cohorts; 80 participants were randomly assigned in a 1:1 ratio to receive 10 Hz rTMS or sham treatment on the bilateral dorsolateral prefrontal cortices (DLPFC) for 4 consecutive weeks. The patients were assessed by ECAS and ALSFRS‐R scales. The Zarit care burden scale was administered to caregivers of ALS patients. The primary outcome measured was the rate of decline in the total ECAS score between pretreatment, 6 months post‐treatment, and 12 months post‐treatment. Secondary outcomes included the group difference in the slope of the Zarit score, ALSFRS‐R total score, and the neurofilament light chain plasma levels.

**Results:**

The ECAS total score in the intention‐to‐treat population significantly changed from 79.74 ± 6.39 to 81.98 ± 6.51 and 79.22 ± 6.50 with rTMS intervention at the 6‐month and 12‐month follow‐ups, respectively (*p* = 0.031, *p* = 0.042). The Zarit score also significantly decreased from 57.65 ± 3.42 to 52.24 ± 3.34 and 56.42 ± 3.41 at the 3‐month and 6‐month post‐treatment time points, respectively (*p* = 0.003, *p* = 0.014). No significant differences were observed between the groups for other secondary endpoints. However, there was a trend of decreasing NF‐L level rates in the treatment group over the first 6 months' follow‐up.

**Conclusions:**

rTMS could yield short‐term positive effects on the ALS patients subgroup with cognitive impairment and alleviate caregivers' burden. No improvement was observed in the severity of ALS and ALS plasma biomarkers.

## Graphical abstract

Study design comparing the treatment and sham groups. The treatment group received 4 weeks of rTMS on the DLPFC (5 days per week), while the sham group underwent sham stimulation. Assessments were conducted at baseline (T0), 4 weeks (T1), 3 months (T2), 6 months (T3), and 12 months (T4), measuring ECAS, Zarit, ALSFRS‐R scores, and NF‐L levels to evaluate group differences.

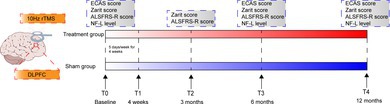

## 1 Introduction

Amyotrophic lateral sclerosis (ALS) is a neurodegenerative disease characterized by the progressive loss of both upper and lower motor neurons. This includes speech articulation problems, difficulty swallowing, muscle atrophy, hyperactive tendon reflexes, and increased muscle tone. The average life expectancy is 2–3 years, with the primary cause of death being respiratory failure due to the involvement of the diaphragm muscle. However, there is still a lack of effective treatments and medications in clinical settings. The high mortality rate and increasing incidence of ALS have a significant impact on public health. However, an increasing number of studies and evidence indicate that some ALS patients exhibit varying degrees of cognitive impairment and behavioral changes, with the severity in some reaching the criteria for frontotemporal dementia (FTD) [1]. Both FTD and ALS are considered part of the same clinical disease spectrum, and cognitive and behavioral disorders not only increase the clinical heterogeneity and caregiver burden in ALS patients but are also potential risk factors for their prognosis.

Recent clinical studies and preliminary work by our team have further found that ALS patients with cognitive or behavioral impairments have a significantly rapid disease progression and shorter survival time, resulting in a poorer prognosis compared to those without such impairments [2].

Repetitive transcranial magnetic stimulation (rTMS) is a noninvasive, safe, and effective nonpharmacological treatment that involves using pulsed magnetic fields to influence central nervous system activity, altering the membrane potential of brain cortical neurons, affecting cellular metabolism within the brain, and modifying brain plasticity. Its safety and widespread use in treating cognitive dysfunctions associated with Alzheimer's disease have been confirmed in multiple clinical studies [3].

The dorsolateral prefrontal cortex (DLPFC) is responsible for executive functions, working memory, abstraction, emotional processing, and inhibition. Clinical studies have shown that high‐frequency rTMS treatment of the DLPFC can significantly improve cognitive dysfunction, including those associated with Alzheimer's disease, and numerous studies have validated its safety. Research indicates that patients with frontotemporal dementia have reduced perfusion and metabolism in the bilateral DLPFC, and functional MRI studies have shown significant functional declines in the DLPFC of ALS patients with executive dysfunctions compared to healthy controls [4]. Furthermore, a prospective study has shown that high‐frequency rTMS can improve cognitive and behavioral functions in patients with FTD [5]. However, current evidence is insufficient to determine whether high‐frequency rTMS on the DLPFC can improve cognitive dysfunctions in ALS patients or if improving these dysfunctions in ALS patients could further slow the progression and severity of the disease, as well as reduce the burden on caregivers.

Therefore, we conducted a randomized clinical trial to evaluate the effect of rTMS on ALS patients with cognitive impairment. This study aimed to explore how rTMS might enhance cognitive function in ALS, potentially easing caregiver burden, reducing severity, and altering plasma biomarkers associated with ALS.

## 2 Material and Methods

### 2.1 Study Design and Participants

A total of 80 ALS patients were recruited from Shanghai Sixth People's Hospital and Shanghai Mental Health Center. Clinical features were collected from ALS patients at the Neuro‐Muscular Specialist Outpatient Clinic of Shanghai Sixth People's Hospital and Shanghai Mental Health Center. The data included family history of ALS or FTD (specifying whether it was sporadic or familial ALS or FTD), gender, age, educational level, time of onset, site of onset, severity scores from the revised ALS Functional Rating Scale [6], and treatment with medications such as riluzole and edaravone. Forced vital capacity was measured in all ALS patients by using a spirometer. Cognitive and behavioral disorders were evaluated using the Chinese version of the ECAS (Edinburgh Cognitive and Behavioural ALS Screen), adhering to the ECAS cognitive scale guidelines [7].

### 2.2 Participant Selection Criteria

**FIGURE 1:**
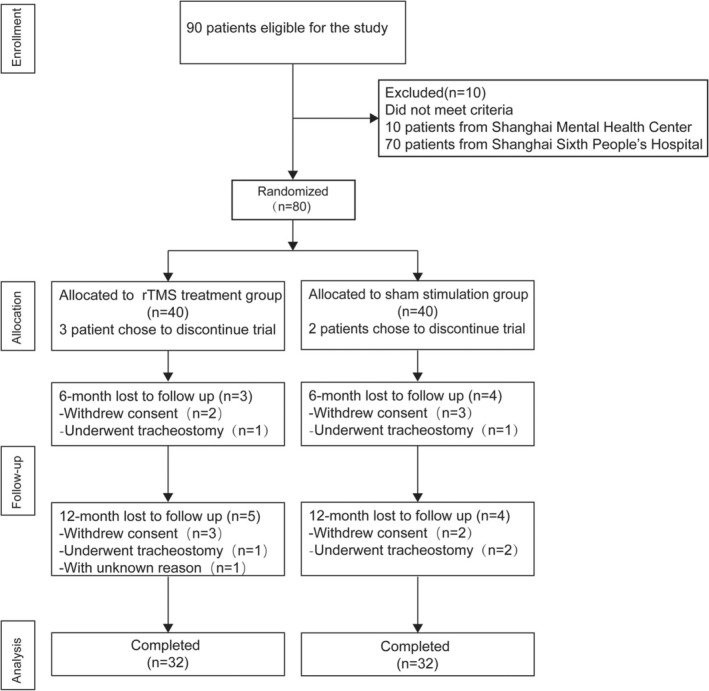
Flowchart of patient recruitment.

Ninety ALS patients were initially eligible for the study. After screening, 80 ALS patients with cognitive or behavioral impairments were enrolled and randomly assigned in a 1:1 ratio to either the rTMS treatment group or the sham stimulation group, with 40 patients in each group. The flow chart of patient recruitment for this trial is illustrated in Figure 1, and the randomization table utilized for participant allocation is provided in Table S7.

All patients met the following inclusion criteria:

1. Age ≥ 18 years old.
2. Must meet the revised El Escorial diagnostic criteria (Brooks et al. Amyotrophic lateral sclerosis and other motor neuron disorders 2000) for a clinically definite or probable diagnosis, showing damage to the upper and lower motor neurons in at least two spinal cord segments.
3. Must display cognitive or behavioral impairments as screened by the ECAS Chinese version (cognitive scale score ≤ 105, behavioral scale score ≥ 1).
4. Forced vital capacity must exceed 75% of the predicted value.
5. Must be capable of providing and signing informed consent.

Patients were excluded if they met any one of the following exclusion criteria:

1. ALS patients who have undergone a tracheostomy.
2. Those with electronic cochlear implants, cardiac pacemakers, metal implants, deep brain stimulators (DBS), or vagus nerve stimulators.
3. Patients with other neurological conditions, including brain tumors, epilepsy, brain trauma, schizophrenia, or other neurodegenerative diseases.
4. ALS patients who were either pregnant or breastfeeding.
5. Patients who have previously received transcranial magnetic stimulation or other cognitive function training therapies.

### 2.3 Interventions

**FIGURE 2:**
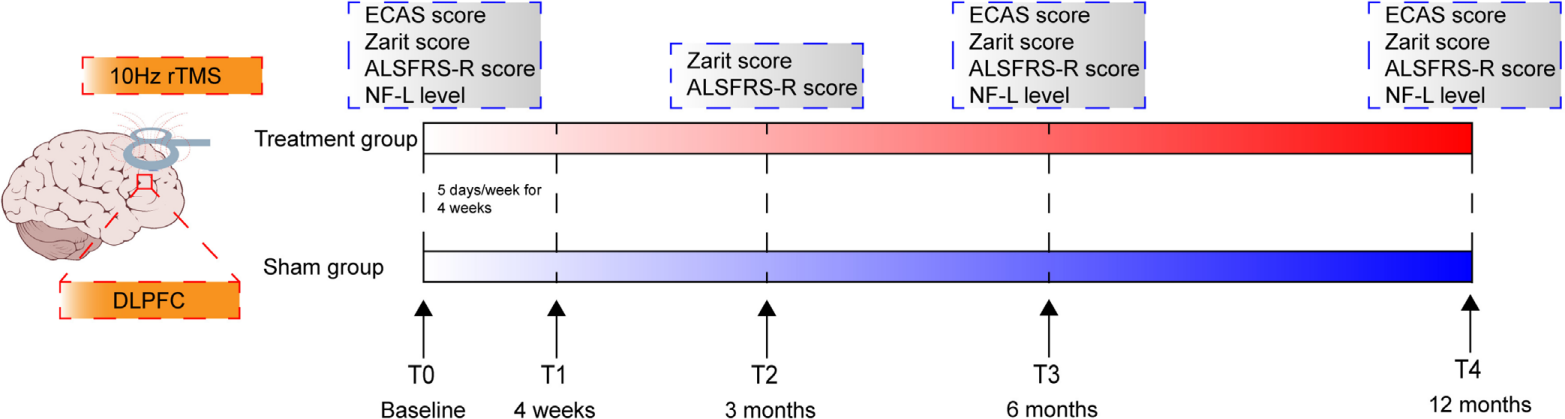
rTMS study design.

ALS patients in both groups underwent rTMS or sham stimulation. The stimulation was applied using the MagPro R30 system (MagVenture, Denmark) to the bilateral dorsolateral prefrontal cortex (DLPFC) with a figure‐of‐eight coil (Cool‐B65，Denmark) at an intensity of 90% personal resting motor threshold level and a frequency of 10 Hz. Each side received 1500 pulses per day (3000 pulses in total), with the treatment course lasting five consecutive days and the entire treatment spanning 4 weeks. The DLPFC was localized using the Beam F3 targeting method [8]. For sham rTMS, the coil was placed at a 90° angle, perpendicular to the participant's scalp, in order to produce the mimic sound and superficial sensation of rTMS without the active effects. A trained physician determined the resting motor threshold level and coil position during the treatment session. 6 and 12 months after the treatment course. The motor evoked potential (MEP) method was used to determine both patient groups' resting motor threshold (MT). The recording electrode was placed at the valley of the abductor pollicis brevis, and the reference electrode was placed at the tendon of the abductor pollicis brevis. With the wrist in a relaxed position, TMS was used to target the corresponding motor cortex area and evoke MEPs. The stimulator intensity was adjusted until MEPs of 50 μV amplitude were produced in at least 5 of 10 trials to determine the MT. Both groups were assessed using the ECAS cognitive and behavioral scales by an independent blinded rater. The caregiver burden was assessed with the Zarit Caregiver Burden Interview [9] at four intervals: before treatment, and 3, 6, and 12 months after treatment. Disease severity in patients was assessed using the Amyotrophic lateral sclerosis functional rating scale‐revised (ALSFRS‐R) at the same above‐mentioned four time points. Prior to the intervention and at the 6‐ and 12‐month follow‐up visits, a routine 5 mL sample of EDTA venous blood was collected from ALS patients. The blood was centrifuged at 3000 rpm to obtain 50 μL of plasma, which was then stored at −80°C until further analysis. In addition, To evaluate blinding integrity, patients will be asked after the treatment session if they believe they received active or sham treatment and their level of certainty. The study design is presented in Figure 2.

### 2.4 Sample Size Determination

PASS software was used to estimate the sample size, which was based on preliminary research data (80% test power (1‐β), a two‐sided alpha value of 5%, and a 20% dropout rate, with a 1:1 ratio of treatment to control group sample size). The minimum sample size was calculated to be 32 participants for the rTMS treatment group and 32 for the sham stimulation control group. Accordingly, a total of 80 patients with cognitive or behavioral disorders associated with ALS were recruited from the neuromuscular disease specialty clinic at Shanghai Sixth People's Hospital and Shanghai Mental Health Center.

### 2.5 Neuropsychological Assessment

All participants were tested with the ECAS, which has a maximum possible score of 136. The cutoff value for cognitive impairment is less than 105. Behavioral impairment was defined as a score of 1 or more. Additionally, caregiver burden was assessed with the Zarit Caregiver Burden Scale, with a higher value indicating more burden and stress. These scales were administered by an independent, blinded clinician.

### 2.6 Ethics Statement

This study was approved by the ethical committee of Shanghai Jiao Tong University Affiliated Sixth People's Hospital (2021‐030‐1). This study was conducted in accordance with the guidelines of Good Clinical Practice and the tenets of the Declaration of Helsinki.

### 2.7 Outcome Measures

The primary endpoint was the change in the ECAS cognitive and behavioral scale between baseline and 6 and 12 months after rTMS treatment. A researcher, blinded to patient groups, assessed the patients using the ECAS cognitive and behavioral scale. Secondary endpoints included the change in ALSFRS‐R scores at 3, 6, and 12 months post‐treatment, as well as caregiver burden scores measured by the Zarit scale at the same intervals for two groups of ALS patients who completed the treatment. The secondary outcome also included the rate of change in the neurofilament light chain (NF‐L) over 6 and 12 months. Plasma levels of NF‐L were measured using an enzyme‐linked immunosorbent assay (ELISA) kit (MBS264177, MyBioSource, Sweden).

### 2.8 Statistical Analysis

Data analysis was performed using IBM SPSS Statistics 20.0 for Windows (IBM Corp., Armonk, NY, USA). Categorical variables were described as frequencies and percentages, while continuous variables with normal distribution were shown as means and standard deviations (SDs). The Kolmogorov–Smirnov test was used to assess the normality of data distribution. For non‐normally distributed continuous variables, medians and interquartile ranges (IQRs) were reported. The Student's t‐test was used to compare two groups with normally distributed data, whereas the Mann–Whitney *U* test was applied to data without normal distribution. The chi‐squared test was used to analyze differences in categorical variables. A linear mixed effect (LME) model was employed to analyze primary and secondary endpoints. This model examined the predictive effects of treatment group, time, and treatment‐by‐time interaction on psychological assessment scores, disease severity scores, and plasma levels of ALS blood biomarkers across groups. An intention‐to‐treat (ITT) approach was utilized to assess the clinical effects of rTMS. A *p* value less than 0.05 was deemed statistically significant. In this model, subjects were considered random effects, and baseline performance scores and the use of edaravone or riluzole were included as covariates. All analyses were Benjamini–Hochberg adjusted for multiple testing. Missing data were handled with multiple imputation in SPSS.

### 2.9 Adverse Event Assessment

At the end of the treatment, patients were asked to self‐report any adverse events at 6 and 12 months post‐treatment by using the SAFTEE scale (a specialized semi‐structured safety assessment tool) for rTMS, developed by Levine J et al. [10]. This standardized interview featured an open‐ended question regarding any adverse events or physical discomfort encountered. If any adverse events or physical discomfort were reported, a standardized adverse event assessment form was filled out. These adverse events were categorized into mild (no impairment), moderate (some impairment or intervention required to prevent impairment), severe (evident impairment and intervention needed), or serious (hospitalization required or significant threat to health/well‐being), including those specific to rTMS, such as headaches, neck pain, dysgeusia, dizziness, and other discomforts. Details about these events' locations, onset, and duration were meticulously compiled into an electronic case report form (eCRF).

## 3 Results

A total of 80 patients met the inclusion criteria and were enrolled in this study between December 2020 and June 2024. Sixty‐four participants completed the treatment session and all follow‐up visits. Demographic information is summarized in Table 1, which indicates that there was no significant difference in age, sex ratio, years of education, and disease duration between the two groups. The ALS onset type was also well balanced.

**TABLE 1:** Comparison of baseline clinical features between groups.

	rTMS group	Sham group	*p*
Total number	32	32	
Males: females (*n*)	19/13	17/15	1.000
Age (years)	59.6 ± 10.30	57.87 ± 11.19	0.575
BMI (kg/m^2^)	23.4 ± 2.8	22.4 ± 2.2	0.549
Years of education (median range)	12 (9, 16)	9 (0, 16)	0.377
Disease duration (days)	595 (208, 2065)	421 (99, 2021)	0.295
ALSFRS‐R	34.2 ± 6.62	38.1 ± 6.37	0.022*
FVC (% predicted)	84.4 ± 19.23	80.43 ± 22.06	0.098
Onset			
Bulbar	5 (15.62%)	4 (12.50%)	1.000
Spinal	27 (84.38%)	28 (87.50%)	

Abbreviations: BMI, body mass index; FVC, forced vital capacity; rTMS, repetitive transcranial magnetic stimulation.

* *p* < 0.05.

### 3.1 Primary Outcome

**FIGURE 3:**
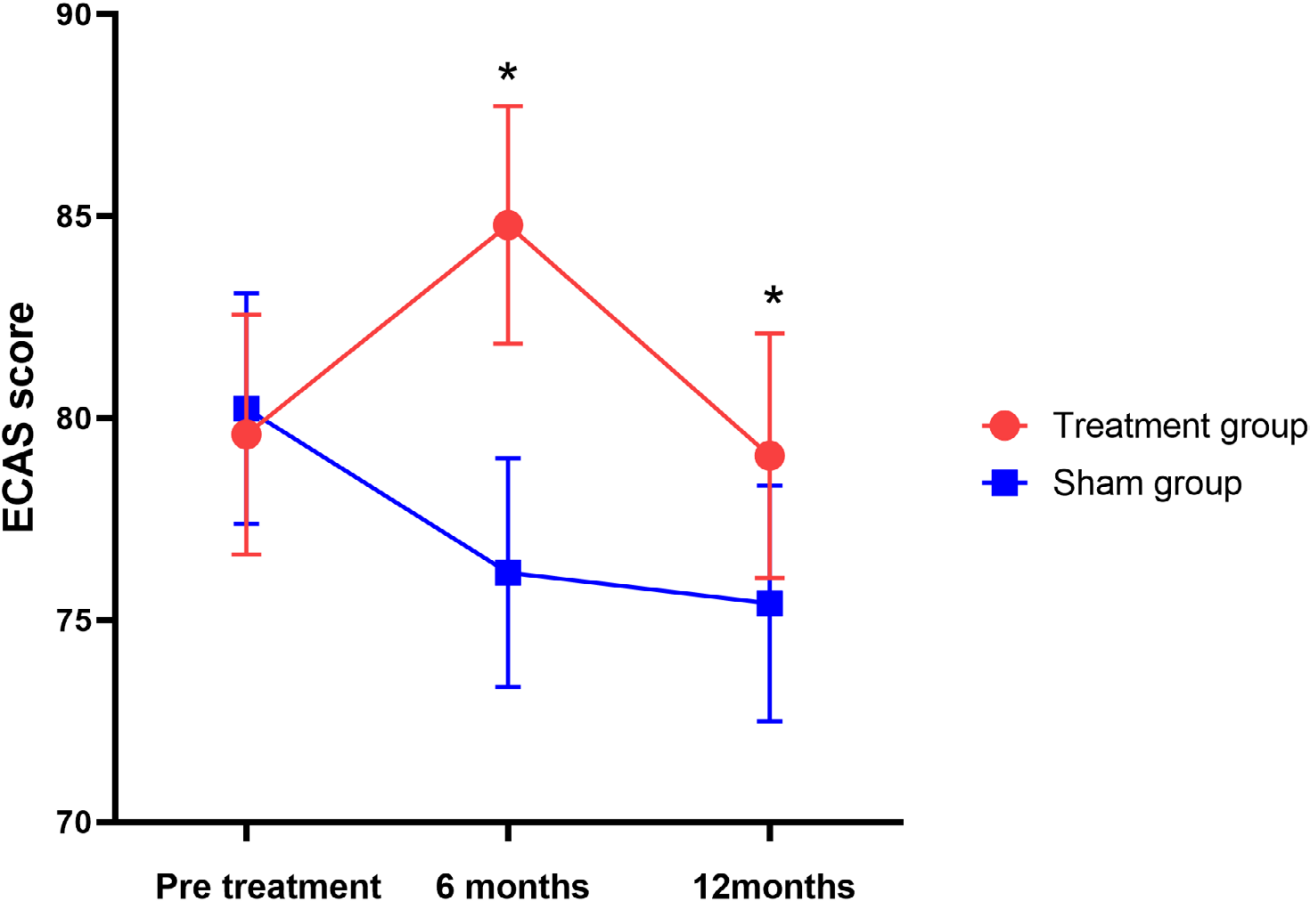
Changes in the ECAS score in the treatment group and sham group. Data are presented as estimated means and standard errors from a linear mixed model. *p < 0.05.

Significant differences were detected between the groups for the primary endpoint based on the LME model. Compared with the sham group, the ECAS total score in the ITT population significantly changed from 79.74 ± 6.39 to 81.98 ± 6.51 and 79.22 ± 6.50 with rTMS intervention at the 6‐month and 12‐month follow‐ups, respectively. The group differences were 6.29 points (95% CI: 4.33–8.26, *p* = 0.031) and 4.30 points (95% CI: 2.20–6.39, *p* = 0.042) (Figure 3, Table 2). Verbal fluency and executive domains exhibited significant differences, while there was no significant difference in the language, memory, and visual‐spatial domains (Figures S9–S13, Table S5). The Supporting Information S1 illustrates the trajectory changes of the ECAS total score for the two groups (Figure S1).

**TABLE 2:** Estimated change over and mean change in primary and secondary outcomes between the rTMS group and sham group.

Outcome	rTMS group (*n* = 32)	Sham group (*n* = 32)		
	Estimated mean	Estimated mean	Estimated mean difference(95% CI)	*p*
*Primary outcome*				
ECAS score				
Baseline	76.26 ± 6.25	79.64 ± 6.39		
6 months after treatment	81.98 ± 6.51	76.16 ± 6.26	6.29 (4.33 to 8.26)	0.031*
12 months after treatment	79.22 ± 6.50	75.39 ± 6.24	4.30 (2.20 to 6.39)	0.042*
*Secondary outcome*				
Zarit score				
Baseline	57.65 ± 3.42	45.78 ± 3.28		
3 months after treatment	52.24. ± 3.34	50.32 ± 3.21	9.94 (5.46 to 14.43)	0.003**
6 months after treatment	56.42 ± 3.41	53.60 ± 3.22	9.04 (3.25 to 14.85)	0.014*
12 months after treatment	60.89 ± 3.65	55.46. ± 3.50	6.44 (−0.11 to 12.89)	0.050
ALSFRS‐R score				
Baseline	33.18 ± 1.48	37.77 ± 1.48		
3 months after treatemnt	31.82 ± 1.51	35.06 ± 1.52	−1.35 (−2.85 to 0.15)	0.075
6 months after treatment	30.27 ± 1.57	34.34 ± 1.52	−0.52 (−3.17 to 2.13)	0.682
12 months after treatment	28.55 ± 2.34	34.33 ± 2.10	1.20 (−5.34 to 7.72)	0.704
Plasma NF‐L level				
Baseline	1.04 ± 0.05	0.86 ± 0.05		
6 months after treatment	1.05 ± 0.06	0.94 ± 0.07	0.07 (0.01 to 0.14)	0.051
12 months after treatment	1.05 ± 0.06	0.93 ± 0.07	0.06 (−0.10 to 0.13)	0.084

*Note:* Parameters were estimated based on a LME model including the time group by time interaction. The model was adjusted for the baseline characteristics and disease duration *p < 0.05, ** *p* < 0.01. Corrected for age, gender, site of onset.

In a sensitivity analysis, correction for the use of edaravone or riluzole gave a result that was in the same trend as the ITT population. There was a significant difference between the two groups at 6 months and 12 months post‐treatment (*p* = 0.036, *p* = 0.041). The estimated mean and standard error are presented in the Supporting Information S1 (Figures S5 and S6, Tables S1 and S2). The cohorts were divided into early‐stage and late‐stage disease groups based on the median of disease duration (568 days). Significant differences were observed between the treatment and sham groups in both subgroups (Figures S7 and S8, Tables S3 and S4).

### 3.2 Secondary Outcome

**FIGURE 4:**
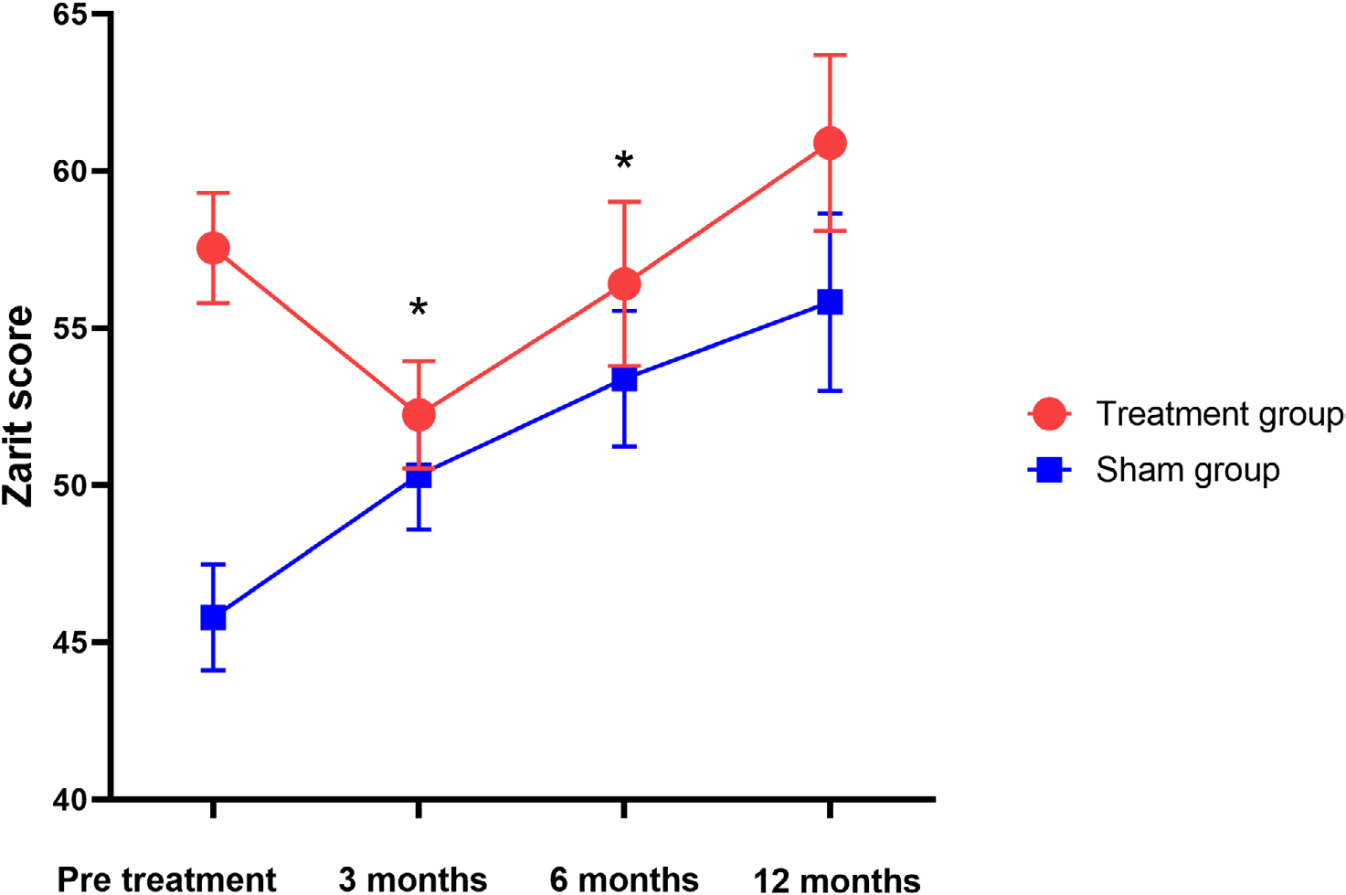
Changes in the Zarit score in the treatment group and sham group. Data are presented as estimated means and standard errors from a linear mixed model. *p < 0.05.

**FIGURE 5:**
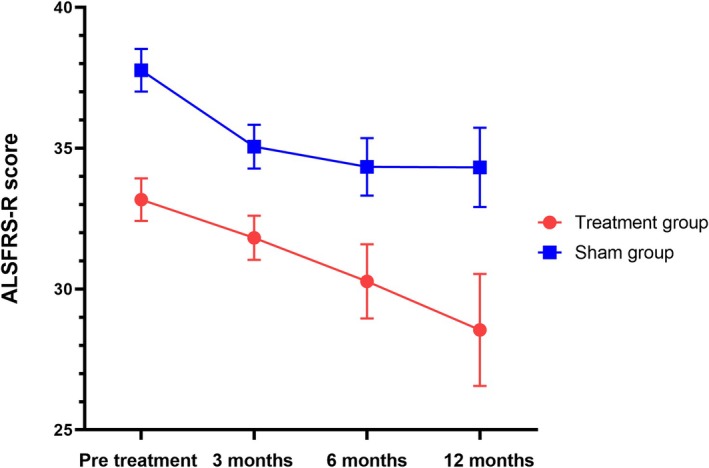
Changes in the ALSFRS‐R score in the treatment group and sham group. Data are presented as estimated mean and standard error from the linear mixed model.

**FIGURE 6:**
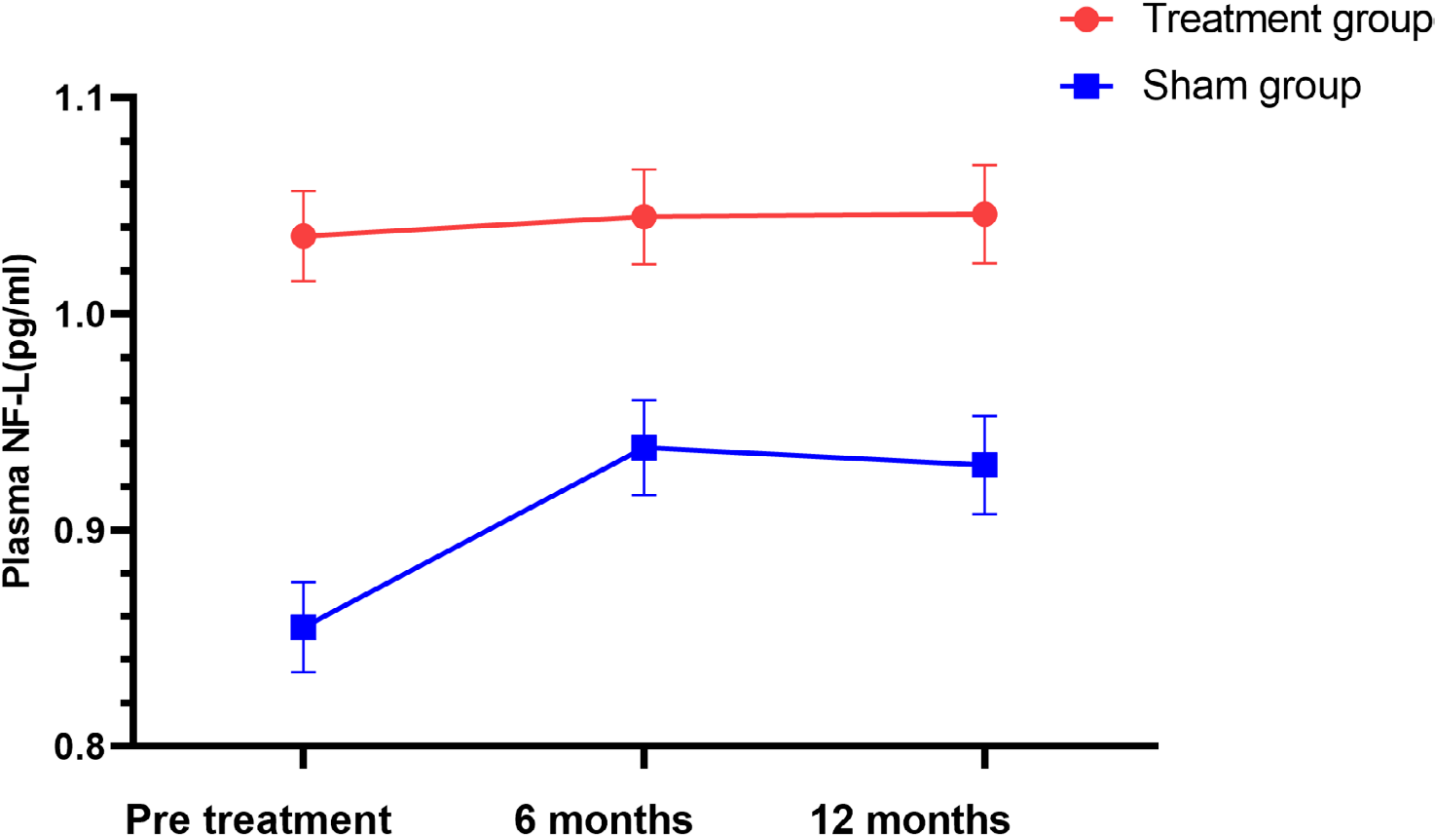
Changes in the level of plasma NF‐L in the treatment group and sham group. Data are presented as the estimated mean and standard error from the linear mixed model.

The results showed a significant trend toward reducing the Zarit score after 3 months and 6 months of intervention. No significant differences were observed between the groups for other secondary endpoints according to the LME model. At 3 and 6 months post‐treatment, the Zarit score significantly decreased from 57.65 ± 3.42 to 52.24 ± 3.34 and 56.42 ± 3.41, respectively, compared to the sham group (*p* = 0.003, *p* = 0.014). The difference between the groups was 9.94 points (95% CI: 5.46–14.43, *p* = 0.003) and 9.04 points (95% CI: 3.25–14.85, *p* = 0.014), as shown in Figure 4 and Table 2. There were no significant changes in the ALSFRS‐R scores over 3, 6, and 12 months between the two groups (*p* = 0.075, *p* = 0.682, *p* = 0.704) (Table 2, Figure 5). No significant differences were noted in the plasma NF‐L levels over 6 and 12 months when comparing treatment and sham stimulation (*p* = 0.051, *p* = 0.084) (Table 2, Figure 6). However, over a 6‐month follow‐up period, the treatment group exhibited a trend of decelerating NF‐L level rate. Changes in the trajectory of Zarit, ALSFRS‐R scores, and plasma NF‐L levels are detailed in the Supporting Information S1 (Figures S2–S4).

**FIGURE 7:**
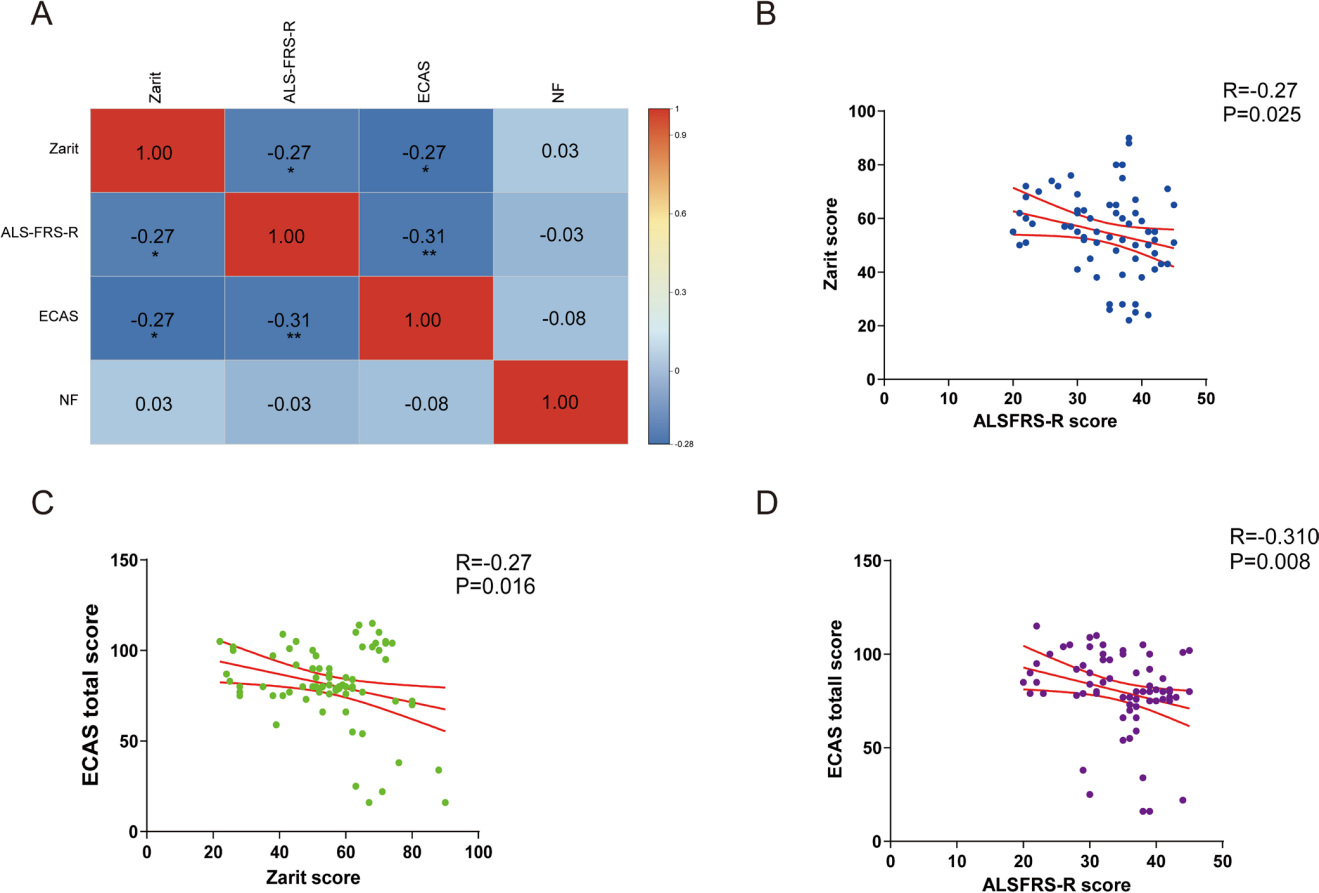
The heat map indicated the correlation analysis between the ECAS score, Zarti score, ALSFRS‐R score, and level of NF‐L (A). A significant negative correlation was found between the Zarit score and the ALSFRS‐R score (*R* = −0.27, *p* = 0.025), as well as between the Zarit score and the ECAS score (*R* = −0.27, *p* = 0.016). Additionally, the ECAS score and ALSFRS‐R score showed a significant correlation (*R* = −0.31, *p* = 0.008). The solid red line and red curve represent the simple linear regression and its 95% confidence interval, respectively. *p < 0.05, **p < 0.01.

Correlation analyses were conducted to examine relationships between ECAS, Zarit, ALSFRS‐R scores, and NF‐L levels (Figure 7). Results revealed significant negative correlations between ECAS and Zarit scores (*p* = 0.016), ECAS and ALSFRS‐R scores (*p* = 0.008), and Zarit and ALSFRS‐R scores (*p* = 0.025). No significant correlation was found between plasma NF‐L and ECAS scores (*p* = 0.329) (Figure S14). The Benjamini–Hochberg–adjusted *p* values were shown in Table S6.

### 3.3 Safety

Only three side‐effect events were reported across both groups. In the rTMS group, one patient reported mild nausea during the initial treatment session. Another patient felt pain at the rTMS application site, which they subsequently tolerated. No further medication was initiated. These incidents were deemed minor (Table 3). No serious adverse events were noted. Additionally, there was no significant disparity in the frequency of adverse events between the treatment and sham groups (*χ* ^2^ test *t*, *p* = 1.00).

**TABLE 3:** Adverse events reported in the two groups.

Adverse events	Treatment group (*n* = 2)	Sham group (*n* = 1)
Headache	0	0
Pain at application site	1	0
Back or Neck pain	0	1
Nausea	1	0
Abnormal sensation	0	0
Other	0	0

## 4 Discussion

TMS is a widely recognized and proven avenue for evaluating and modulating neural excitability and synaptic neuroplasticity in specific brain areas. It is an effective, noninvasive, and promising approach to neuromodulation treatment, enhancing the functional recovery of cortical and neural functions. Several animal models and clinical trials have shown encouraging outcomes in treating cognitive and neurodegenerative diseases [11, 12]. To our knowledge, this is the first randomized clinical study about the effect of rTMS on ALS‐specific subgroup patients with cognitive impairment.

Several studies have shown that the selective hyperexcitability of the primary motor cortex is believed to contribute to motor neuron degeneration in ALS, as observed in human electrophysiological studies [13, 14]. One study showed that the primary cortical hyperexcitability measured by short interval intracortical inhibition (SICI) and the index of excitability (IE) were more pronounced in ALS patients with cognitive impairment. SICI was positively correlated with the level of cognitive impairment [15]. Another study showed that interneuron circuit dysfunction drives cortical hyperexcitability in ALS [16]. The SICI serves as an indicator of GABAergic inhibitory interneuronal activity, likely reflecting dysfunction or degeneration in GABAergic neurotransmission. Hyperexcitability levels are also linked to prognosis, with diminished intracortical inhibition recognized as an independent factor influencing survival [17]. In our study, there was no significant difference between the two groups in terms of MT during the treatment period. Interestingly, the amplitude of the MT was significantly higher in patients exhibiting a high level of upper limb weakness and muscle atrophy. One study has suggested the heterogeneity of cortical dysfunction in ALS patients' different body regions, showing greater cortical hyperexcitability in the upper limbs. In contrast, cortical inexcitability was more evident in the lower limbs and bulbar areas [18].

Previous clinical trial attempts to treat ALS patients have mainly focused on employing rTMS with different frequencies to stimulate the primary motor cortex continuously. Essentially, while low‐frequency rTMS (< 1 Hz) reduces cortical excitability, high‐frequency rTMS (> 10 Hz) increases it. Although previous research has utilized rTMS treatment on the primary motor cortex in ALS patients, there has been no significant impact on the ALSFRS‐R decline rate, or the effects have been minimal [19, 20]. The electromagnetic field generated by rTMS changed neuronal excitability by modulating synaptic plasticity and neurotrophic factors. In animal studies, prior research has demonstrated that high‐frequency rTMS influences the mRNA expression of brain‐derived neurotrophic factor (BDNF) and improves neurons' accurate and precise functioning. This leads to cellular modifications that enhance the excitability and metabolic activity in targeted cortical areas by promoting cortical remapping [21]. BDNF is the potential serum biomarker of ALS with the C9orf72 mutation, while the C9orf72 repeat expansion elevates the risk of cognitive impairment in ALS and ALS‐FTD [22, 23]. Cortical hyperexcitability appears to trigger DNA‐binding protein 43‐kDa (TDP‐43) pathology, which plays a crucial role in ALS patients experiencing cognitive decline [24].

The heterogeneity of brain cortex excitability has also been observed in other cortical areas of the patients' brains. FTD is characterized by prefrontal hypoexcitability [25]. Greater reduction of glucose metabolism and more TDP‐43 deposition were observed in the frontal and parietal regions in ALS‐FTD patients [26]. The prefrontal regions were selected for stimulation in our study based on evidence that these areas play crucial roles in various cognitive processes, such as working and episodic memory, inhibition, monitoring, strategic organization, and planning. Additionally, data suggest that high frequency TMS could stimulate the DLPFC and enhance language capabilities, attention, memory, and other cognitive abilities in healthy individuals [27]. Our study showed that ECAS scores improved in the first 6 and 12 months post‐treatment, and caregiver burden significantly decreased in the first 3 and 6 months post‐treatment. A similar trend was also observed in the ALS specific domain verbal fluency and executive functions associated with DLPFC. The effect of rTMS on the same domains was also shown in the other diseases [28]. rTMS can reduce the caregiver's burden, which is crucial for preserving the caregiver's mental health. However, no change was observed in the ALSFRS‐R score within 12 months post‐treatment. The burden on caregivers continued to increase, with no significant difference noted during the 12‐month follow‐up visits. This suggests that rTMS primarily enhances cognitive performance in a short period. One of the main technical challenges of noninvasive brain stimulation is that rTMS demands time, specialized equipment, and professional staff. The limited “dosage” effect of brain stimulation is crucial for the efficacy of rTMS, as it is restricted to hospital settings and brief treatment durations. The effects gradually diminish after the treatment course. Determining the optimal rTMS intervention duration likely involves a trade‐off. While longer treatment may enhance efficacy, it could also raise dropout rates due to the need for daily clinic visits. Developing simpler, clinically appropriate, and portable equipment is therefore vital to extend the duration of TMS effects and prolong treatment benefits. Furthermore, investigating newly developed rTMS techniques, such as continuous theta burst stimulation (cTBS), is critical to maximize TMS advantages and maintain a persistent effect for ALS patients [29].

Some studies have indicated that cognitive impairment is more prominent in the advanced stage of ALS [30]. No declining trend of cognitive impairment was observed in the follow‐up study [31]. Another study revealed that ALS patients with cognitive impairments experienced more falls than those without [32]. This implies that ALS patients with cognitive impairment require additional support from caregivers, consistent with our study findings. Our research found that the ECAS score was negatively correlated with both the Zarit score and the ALSFRS‐R score. As the disease progresses, the caregivers' burden intensifies. However, the Zarit Burden Interview is subjective, meaning that caregiver stress levels cannot be directly measured in clinical settings and usually depend on self‐reports from caregivers. This suggests that alterations are not measured objectively, making them inappropriate for clinical research settings. Thus, a more precise and objective scale or monitoring system needs to be developed. The ALSFRS‐R and ECAS scores reflect the motor and cognitive functions of ALS. These two scales were also predictive of the survival of patients and the caregiver's quality of life (QoL), Enhancing these two scales could improve patient outcomes and reduce caregivers' psychological burden [33].

Several studies have indicated that high‐frequency rTMS could heighten motor cortex excitability and amplify glutamatergic toxicity, potentially elevating seizure‐related adverse event risks. Yet, recent retrospective clinical trials have shown that high‐frequency rTMS does not notably increase seizure incidences compared to low‐frequency rTMS [34, 35]. Adhering strictly to rTMS operational procedures, intensity, and frequency parameters and appropriately selecting patients for treatment can effectively prevent seizures. In our study, no cases of epilepsy or seizure‐related adverse events were reported. rTMS protocols are well tolerated and safe in accordance with the current safety guidelines. We also used the beam F3 method, known for its superior precision and reliability in identifying the target site compared to the conventional 5.5 cm method, avoiding the side effects of stimulation of the premotor cortex [36]. The figure‐of‐eight coil was more advantageous in delivering localized and focal brain stimulation compared with other coils [37].

In this study, we utilized the ECAS cognitive impairment screening instrument, developed specifically for ALS patients. This tool is tailored to accommodate the physical disabilities that may obscure cognitive performance in ALS cases. In longitudinal research, the repeated use of cognitive assessments is associated with practice effects (PE), which differ from random performance variations and denote increasing familiarity with the test content or procedures. Despite employing different versions of the tests, PE cannot be entirely eliminated. In this study, the ECAS cognitive assessment was followed up 6 months after rTMS treatment. This interval aims to prevent participants' PE and effectively evaluate the effect of rTMS; hence, the choice of a gap of more than 6 months for the follow‐up visit has also been applied in other research [38]. We utilized a recommended cutoff value to screen patients for cognitive impairment (CI), with the subsequent step being the classification of these patients into various levels of cognitive impairment for further exploration in future clinical trials. Our research demonstrated that there is no significant correlation between ECAS and ALSFRS‐R scores, indicating that cognitive performance remains relatively stable over time. Another study suggested that early changes, typically in verbal fluency, are likely to evolve with a faster clinical trajectory characterized by rapid progression within the motor system [39]. Additional 24‐month or 36‐month follow‐up visits are necessary to clarify this.

NF‐L is an emerging biomarker for ALS. UMN‐predominant ALS patients have higher levels of NF‐L compared with other phenotypes [40]. Plasma NF‐L levels correlated with FTD severity indicators and served as a promising diagnostic and prognostic biomarker for FTD [41]. In this study, we did not find a significant difference between the two groups with respect to the level of NF‐L over time after rTMS intervention. One study showed that rTMS could reduce the increased rate of NF‐L caused by alcohol use [42]. NF‐L levels were not significantly associated with the ECAS score, consistent with other studies' observations [43]. TDP‐43 protein plays a crucial role in binding the mRNA of NF‐L and stabilizing NF‐L proteins. Restoring TDP‐43 proteinopathy might also rejuvenate neurofilament protein synthesis in the ALS/FTD mouse model [44]. However, whether rTMS can regulate TDP‐43 protein and subsequently enhance NF‐L function remains to be determined. A study showed that transcranial direct current stimulation (tDCS) on the primary motor cortex of ALS patients significantly improved global strength, reduced caregiver burden, enhanced quality of life scores, and decreased serum NF‐L levels [45]. Interestingly, our study showed a trend of decreasing NF‐L level rates in the treatment group over a 6‐month follow‐up, though it was not statistically significant. Due to the heterogeneity of ALS, the biomarker NF‐L level reflects both motor neuron and cognitive functions. Combining various noninvasive brain stimulations targeting different stimulating areas with different parameters may enhance therapeutic effects for ALS with heterogeneous clinical features.

Our results should be considered in the context of several limitations. One limitation is that not all the participants have had genetic testing results available. The further relationship between genotype and the effect of the rTMS still needs further exploration. Second, a key methodological issue in rTMS trials is the challenge of blinding researchers. Only participants and raters should be blinded to treatment allocation. A meta‐analysis indicated no difference in blinding success between real and sham coil strategies, supporting the need to blind only participants and raters [46]. Participants who exhibited clinical improvement at the end of the treatment were more likely to believe they had received genuine rTMS treatment and guess their treatment allocation. This correlation is likely due to the greater therapeutic efficacy of active interventions compared to sham or placebo treatments. The main limitation of this trial is the small participant number, with some unable to complete the follow‐up due to physical disabilities. Therefore, follow‐up results should be interpreted cautiously, and future trials should involve more patients.

## 5 Conclusion

To our knowledge, this is the first and only randomized controlled trial to date to show that rTMS could improve ALS with cognitive impairment and reduce carer burden. This means rTMS may improve cognitive performance in ALS in the first 12 months post‐intervention. It can also reduce the caregiver's burden in the first 6 months post‐treatment, suggesting that rTMS should be initiated when ALS subgroup patients are diagnosed with cognitive impairment.

## Author Contributions

Wensi Zheng and Xiaojie Zhang wrote the main manuscript. All authors reviewed the manuscript. The corresponding author, Zhouwei Xu, had final responsibility for the decision to submit for publication. The authors thank the patients who participated in this clinical trial.

## Conflicts of Interest

The authors declare no conflicts of interest.

## Supporting information


Data S1.

## Data Availability

The data that support the findings of this study are available from the corresponding author upon reasonable request.
