# Supplementary material for: The Effect of Repetitive Transcranial Magnetic Stimulation of the Dorsolateral Prefrontal Cortex on the Amyotrophic Lateral Sclerosis Patients With Cognitive Impairment: A Double‐Blinded, Randomized, and Sham Control Trial
Source: CNS Neurosci Ther. 2025 Mar 18;31(3):e70316. doi: 10.1111/cns.70316 (PMC11915350; doi:10.1111/cns.70316)
Supplement: Supplementary file 1 — Data S1. [file CNS-31-e70316-s001.docx]

Supplementary materials


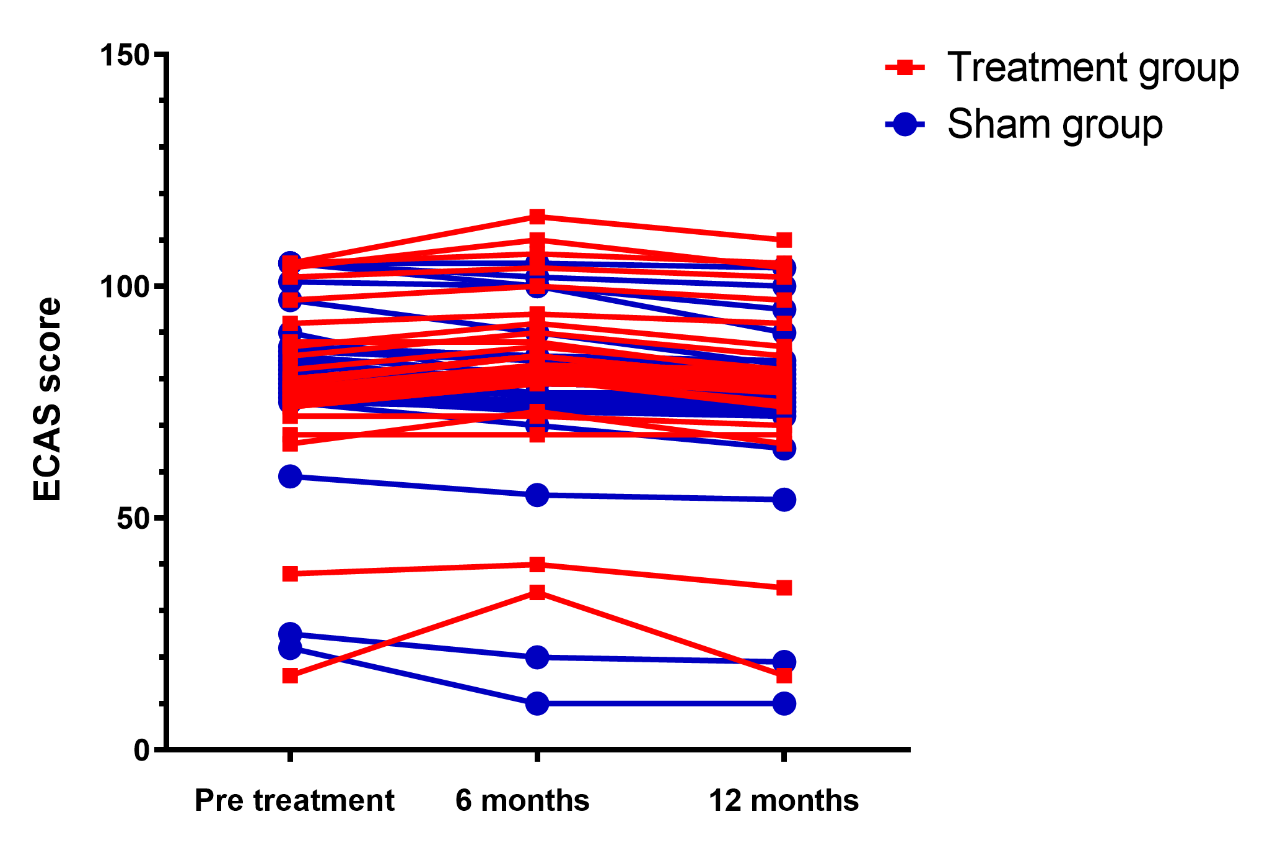


**Figure 1: Changes of ECAS score in the two different groups. The longitudinal curves were plotted in every patient according to different group.**

**
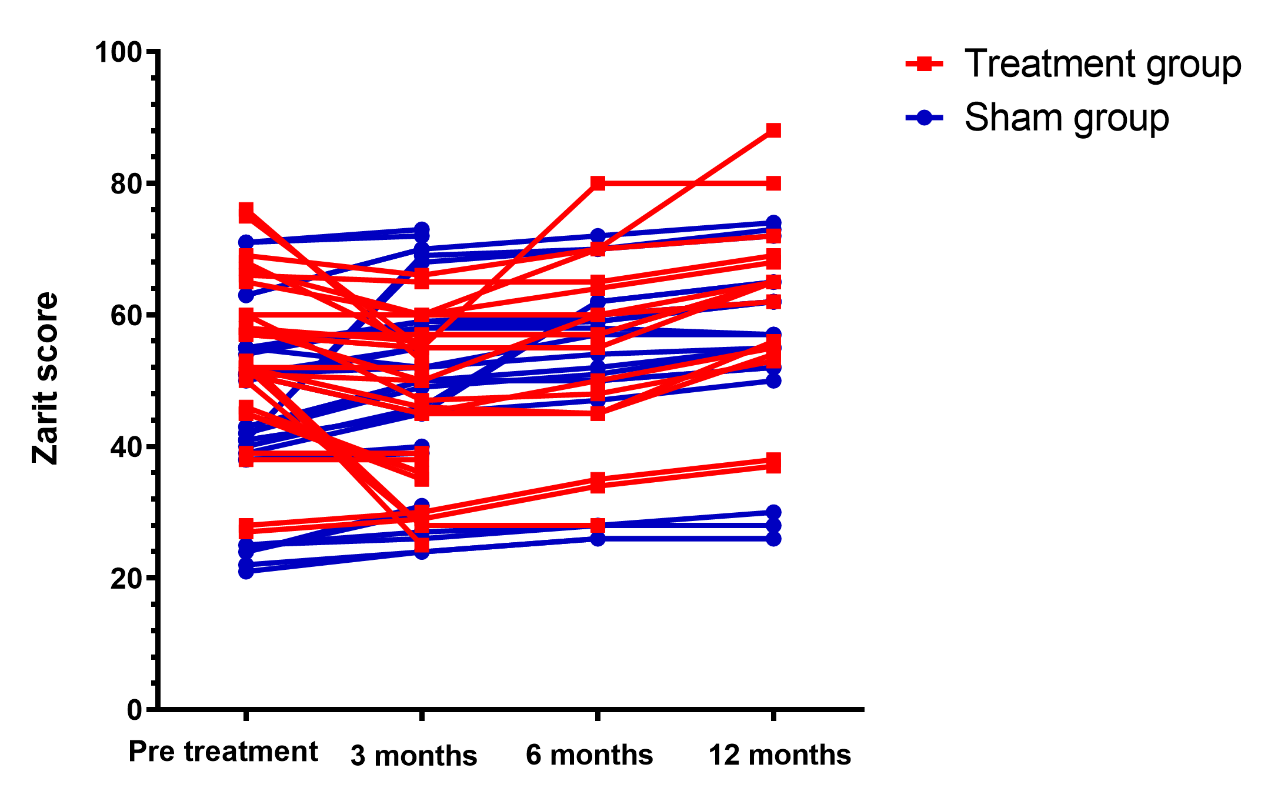
**

**Figure 2: Changes of Zarit score in the two different groups. The longitudinal curves were plotted in every patient according to different group**


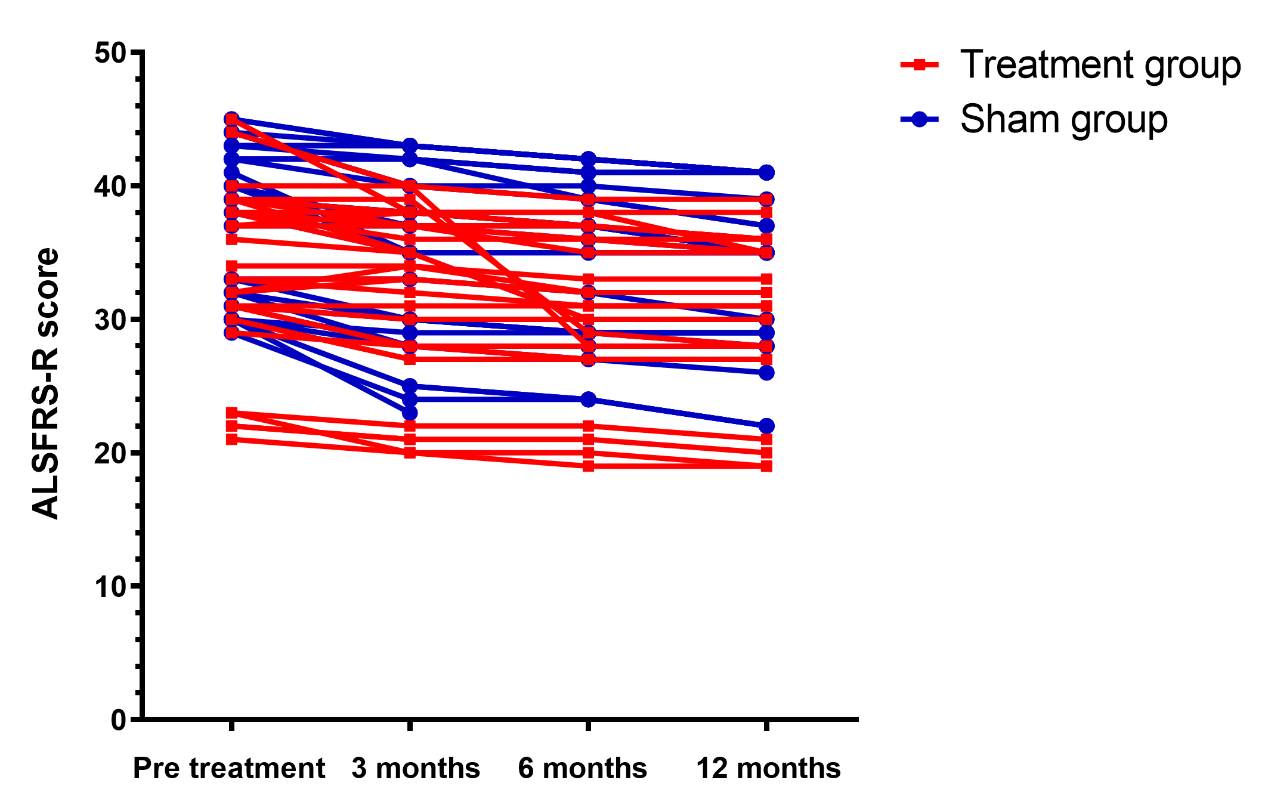


**Figure 3:Changes of ALSFRS-R score in the two different groups. The longitudinal curves were plotted in every patient according to different group.**


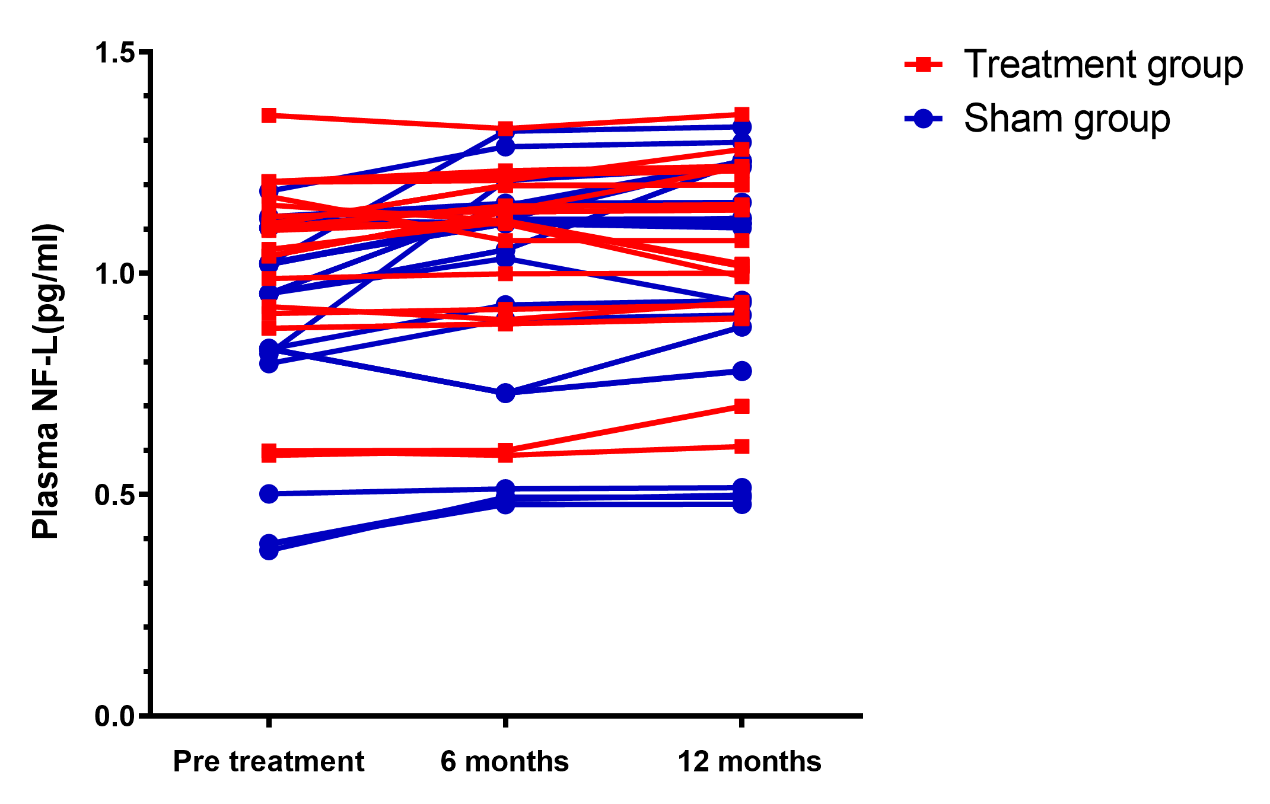


**Figure 4: Changes of Levels of Plasma NF-L in the two different groups. The longitudinal curves were plotted in every patient according to different group.**


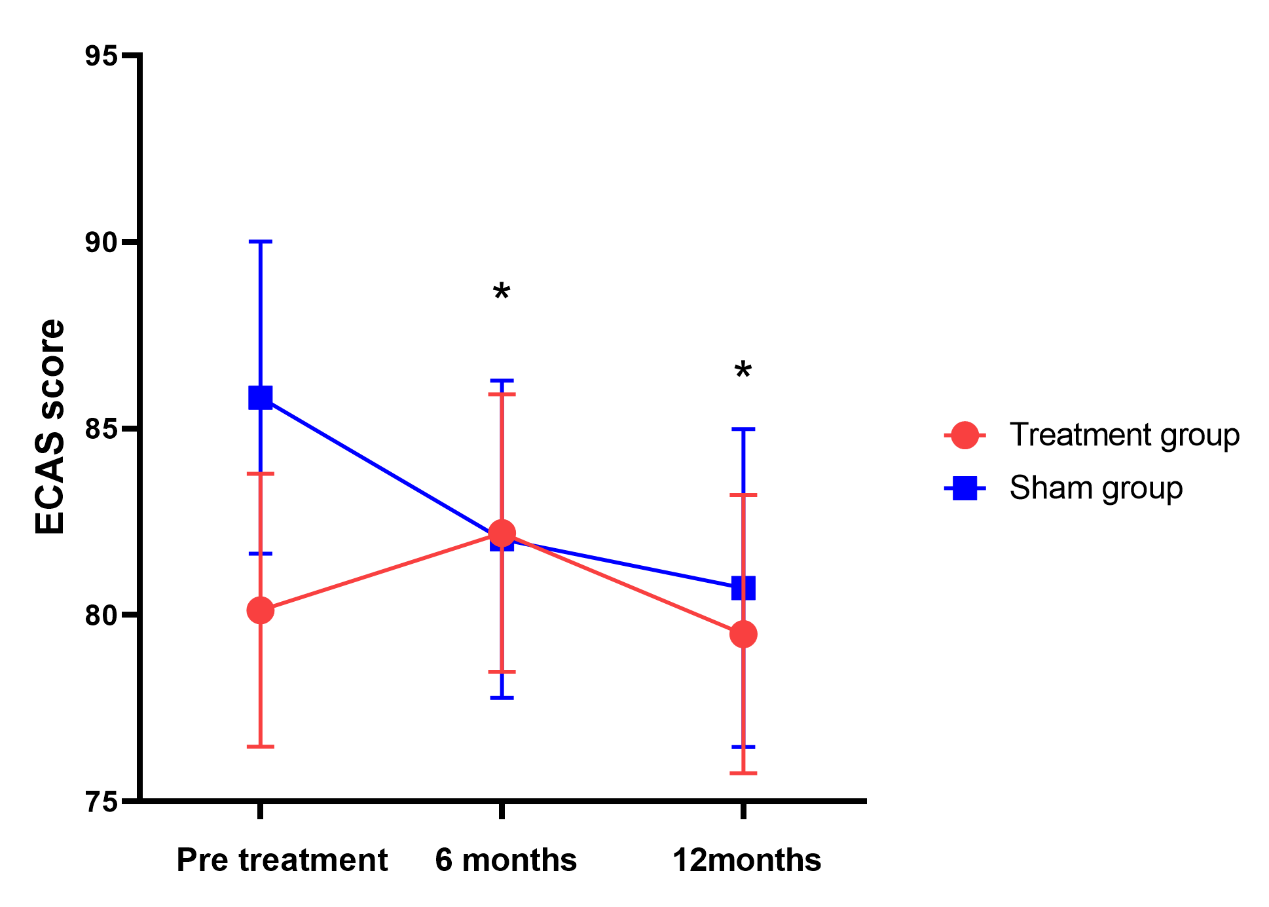


**Figure 5: Changes in the ECAS score in the Treatment group and Sham group corrected for riluzole used**

**Data are presented as estimated mean and standard error from a linear mixed model. *P**<**0.05, **P**<**0.01.**

**Table 1：Data for the changes in the ECAS score in the Treatment group and Sham group corrected for riluzole used**

|  | Time points | Estimated mean | Standard error | Upper 95%  CI | Lower 95% CI | P value |
| --- | --- | --- | --- | --- | --- | --- |
| Sham treatment | Baseline | 85.827 | 8.135 | 69.19 | 102.464 | N/A |
| Sham treatment | 6 month | 82.027 | 8.289 | 65.075 | 98.979 | N/A |
| Sham treatment | 12 month | 80.727 | 8.294 | 63.766 | 97.688 | N/A |
| rTMS treatment | Baseline | 80.129 | 7.118 | 65.573 | 94.686 | N/A |
| rTMS treatment | 6 month | 82.201 | 7.244 | 67.39 | 97.012 | 0.032* |
| rTMS treatment | 12 month | 79.487 | 7.248 | 64.668 | 94.305 | 0.046* |


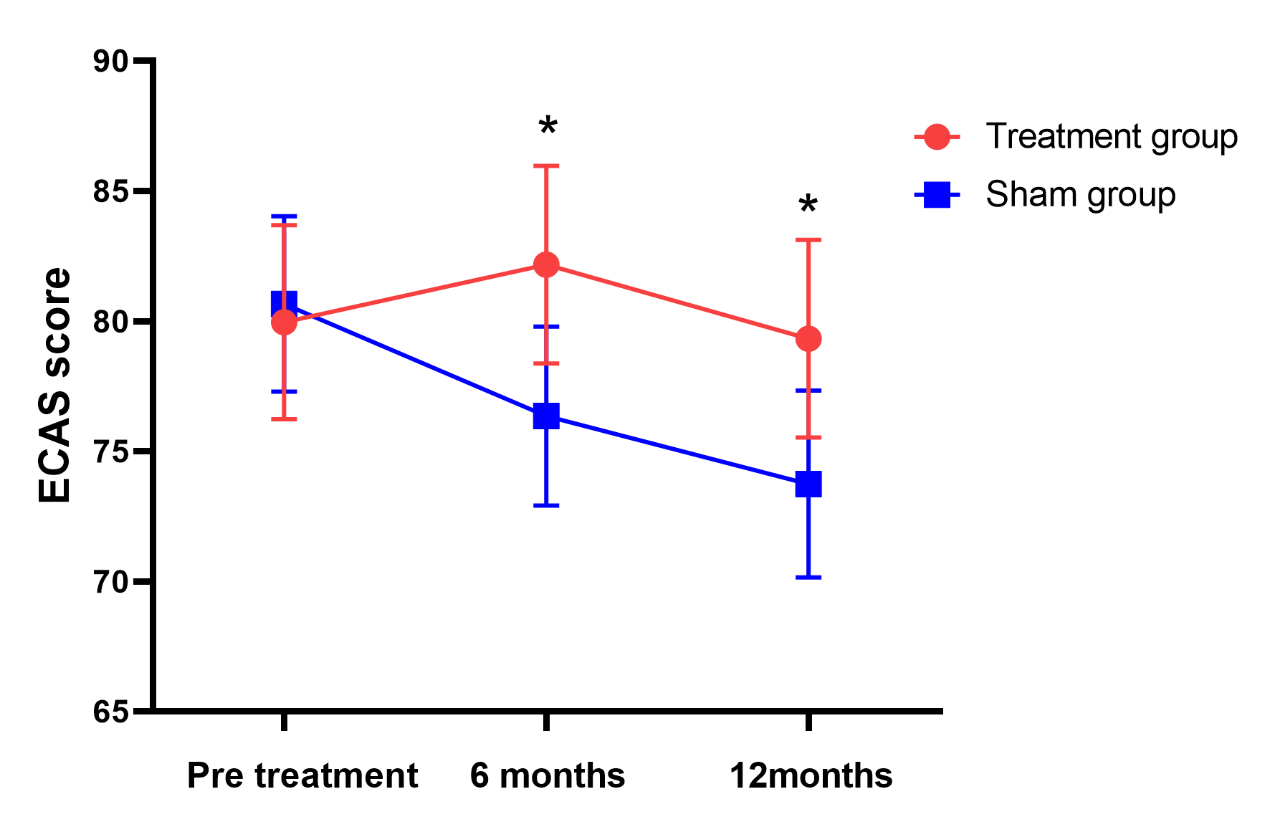


**Figure 6：Changes in the ECAS score in the Treatment group and Sham group corrected for edaravone used**

**Data are presented as estimated mean and standard error from a linear mixed model. *P**<**0.05, **P**<**0.01.**

**Table 2：Data for the changes in the ECAS score in the Treatment group and Sham group corrected for edaravone used**

|  | Time points | Estimated mean | Standard error | Upper 95%  CI | Lower 95% CI | P值 |
| --- | --- | --- | --- | --- | --- | --- |
| Sham treatment | Baseline | 80.657 | 6.542 | 67.276 | 94.039 | N/A |
| Sham treatment | 6 month | 76.345 | 6.691 | 62.659 | 90.031 | N/A |
| Sham treatment | 12 month | 73.741 | 6.987 | 18.546 | 128.936 | N/A |
| rTMS treatment | Baseline | 79.96 | 7.251 | 65.131 | 94.789 | N/A |
| rTMS treatment | 6 month | 82.175 | 7.404 | 67.035 | 97.314 | 0.042* |
| rTMS treatment | 12 month | 79.317 | 7.383 | 64.222 | 94.413 | 0.048* |


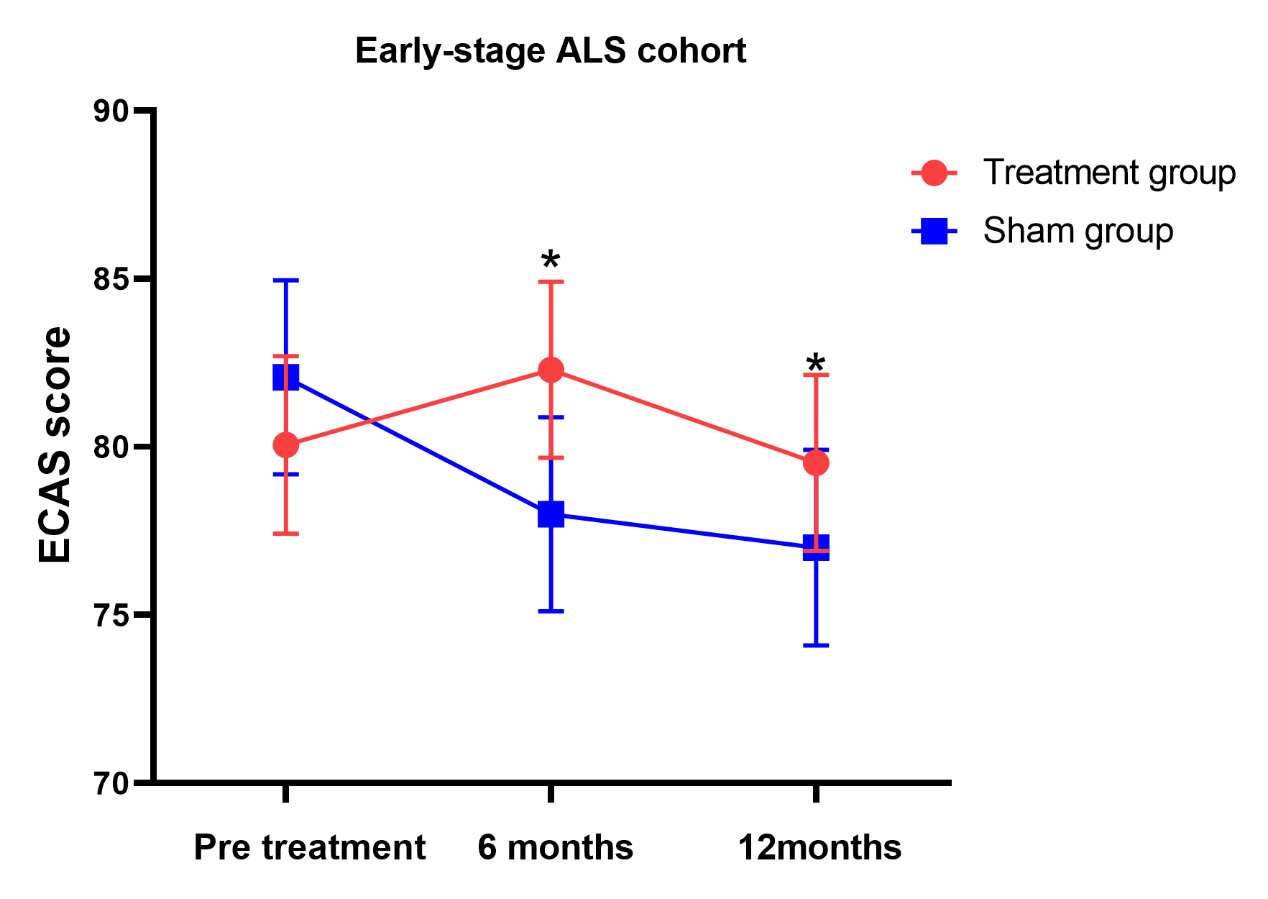


**Figure 7：Changes in the ECAS score in the Treatment group and Sham group in the early stage cohort(disease duration≤568 days)**

**Data are presented as estimated mean and standard error from a linear mixed model. *P**<**0.05, **P**<**0.01.**

**Table 3:** **Data for the changes in the ECAS score in the Treatment group and Sham group in the early stage cohort**

|  | Time points | Estimated mean | Standard error | Upper 95%  CI | Lower 95% CI | P值 |
| --- | --- | --- | --- | --- | --- | --- |
| Sham treatment | Baseline | 82.071 | 6.218 | 69.360 | 94.783 | N/A |
| Sham treatment | 6 month | 78.357 | 6.171 | 65.740 | 90.974 | N/A |
| Sham treatment | 12 month | 77.571 | 6.175 | 64.947 | 90.196 | N/A |
| rTMS treatment | Baseline | 80.059 | 5.645 | 68.518 | 91.599 | N/A |
| rTMS treatment | 6 month | 82.294 | 5.603 | 70.840 | 93.749 | 0.042* |
| rTMS treatment | 12 month | 79.529 | 5.607 | 68.068 | 90.991 | 0.048* |


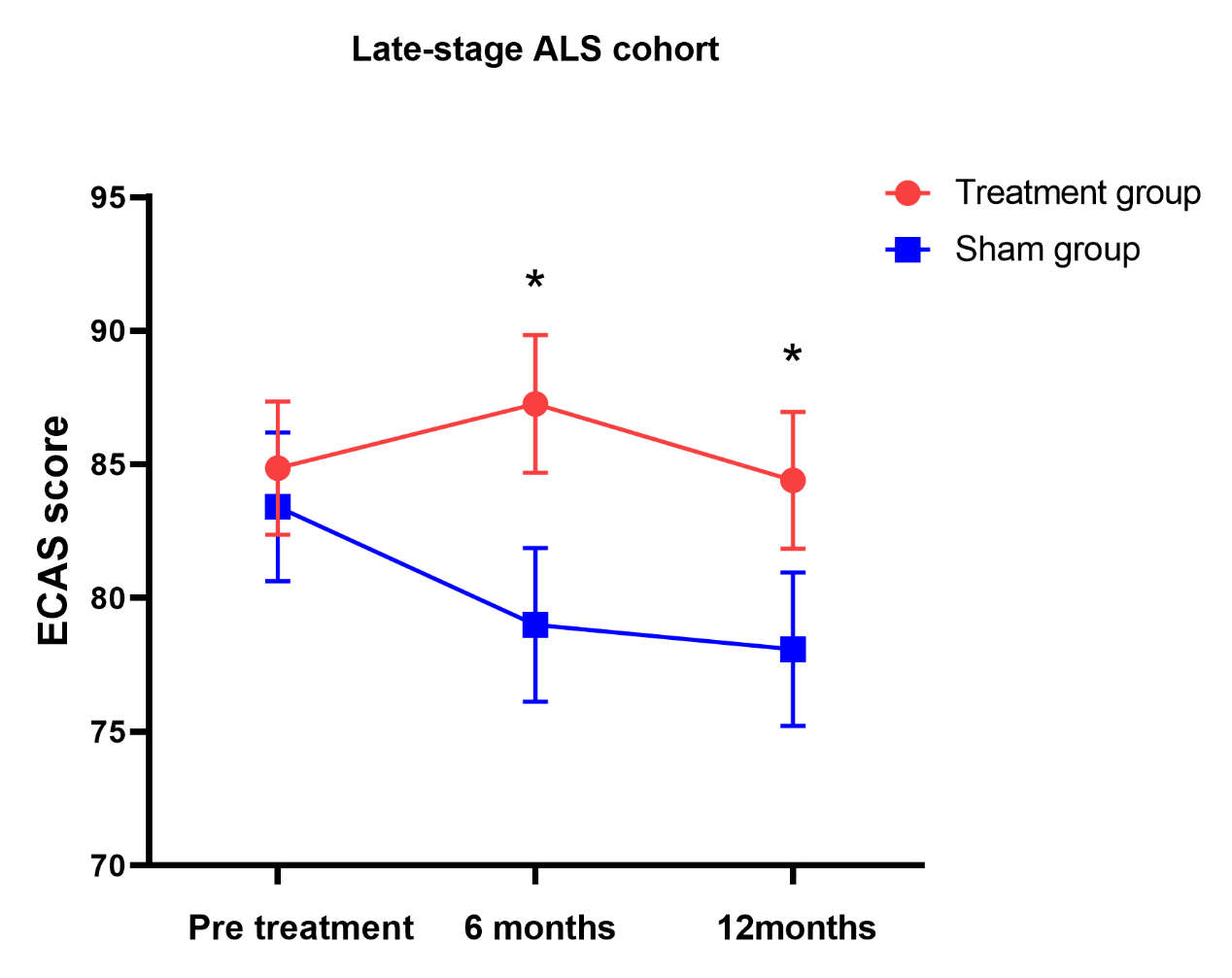


**Figure 8：Changes in the ECAS score in the Treatment group and Sham group in the late-stage cohort（disease duration**>**568 days）**

**Data are presented as estimated mean and standard error from a linear mixed model. *P**<**0.05, **P**<**0.01.**

**Table 4: Data for the changes in the ECAS score in the Treatment group and Sham group in the early stage cohort**

|  | Time points | Estimated mean | Standard error | Upper 95%  CI | Lower 95% CI | P值 |
| --- | --- | --- | --- | --- | --- | --- |
| Sham treatment | Baseline | 83.417 | 5.959 | 71.144 | 95.689 | N/A |
| Sham treatment | 6 month | 79.000 | 6.148 | 66.337 | 91.663 | N/A |
| Sham treatment | 12 month | 78.083 | 6.139 | 65.441 | 90.726 | N/A |
| rTMS treatment | Baseline | 84.867 | 5.330 | 73.890 | 95.844 | N/A |
| rTMS treatment | 6 month | 87.267 | 5.499 | 75.941 | 98.593 | 0.042* |
| rTMS treatment | 12 month | 84.400 | 5.490 | 73.092 | 95.708 | 0.048* |


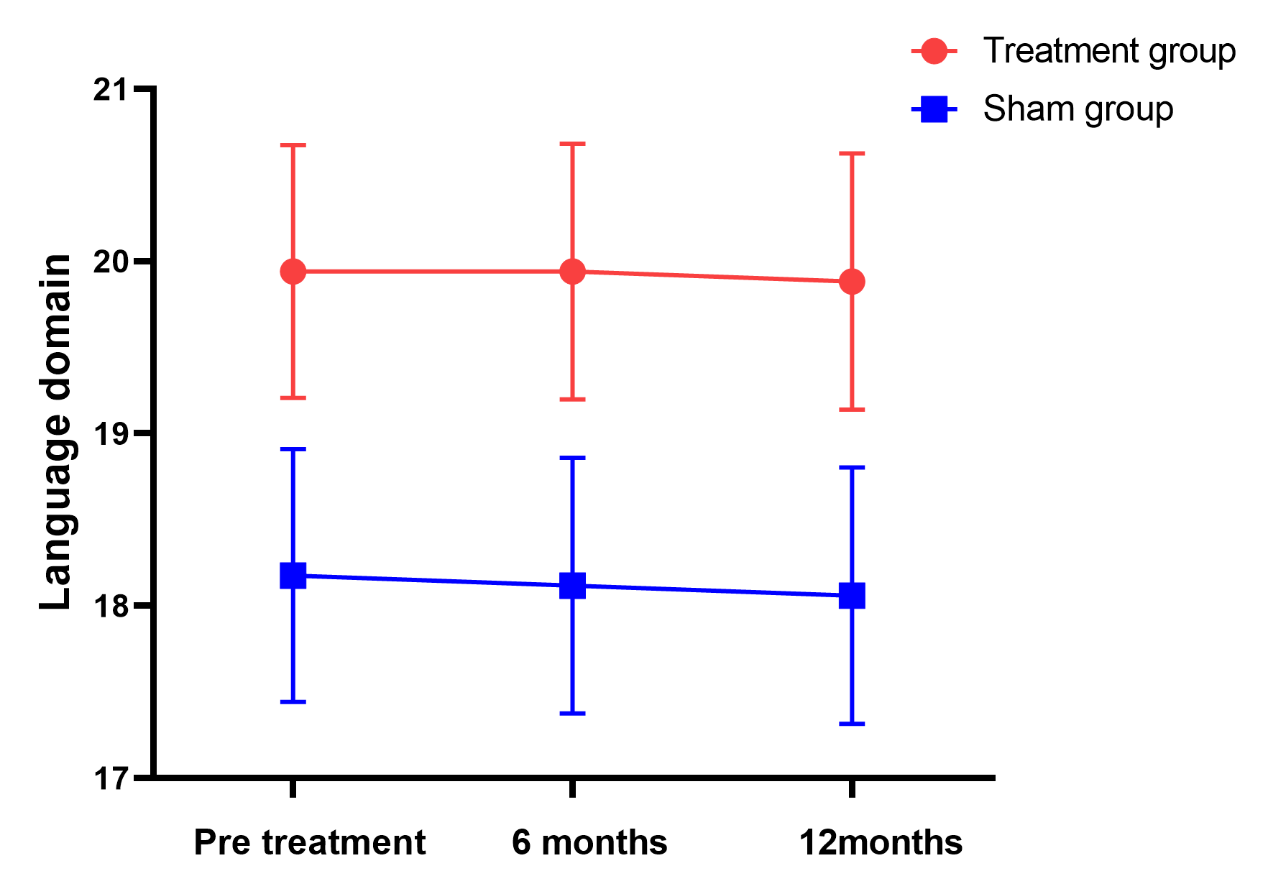


**Figure 9：Changes in the ECAS language domain in the Treatment group and Sham group**

**Data are presented as estimated mean and standard error from a linear mixed model. *P**<**0.05, **P**<**0.01.**


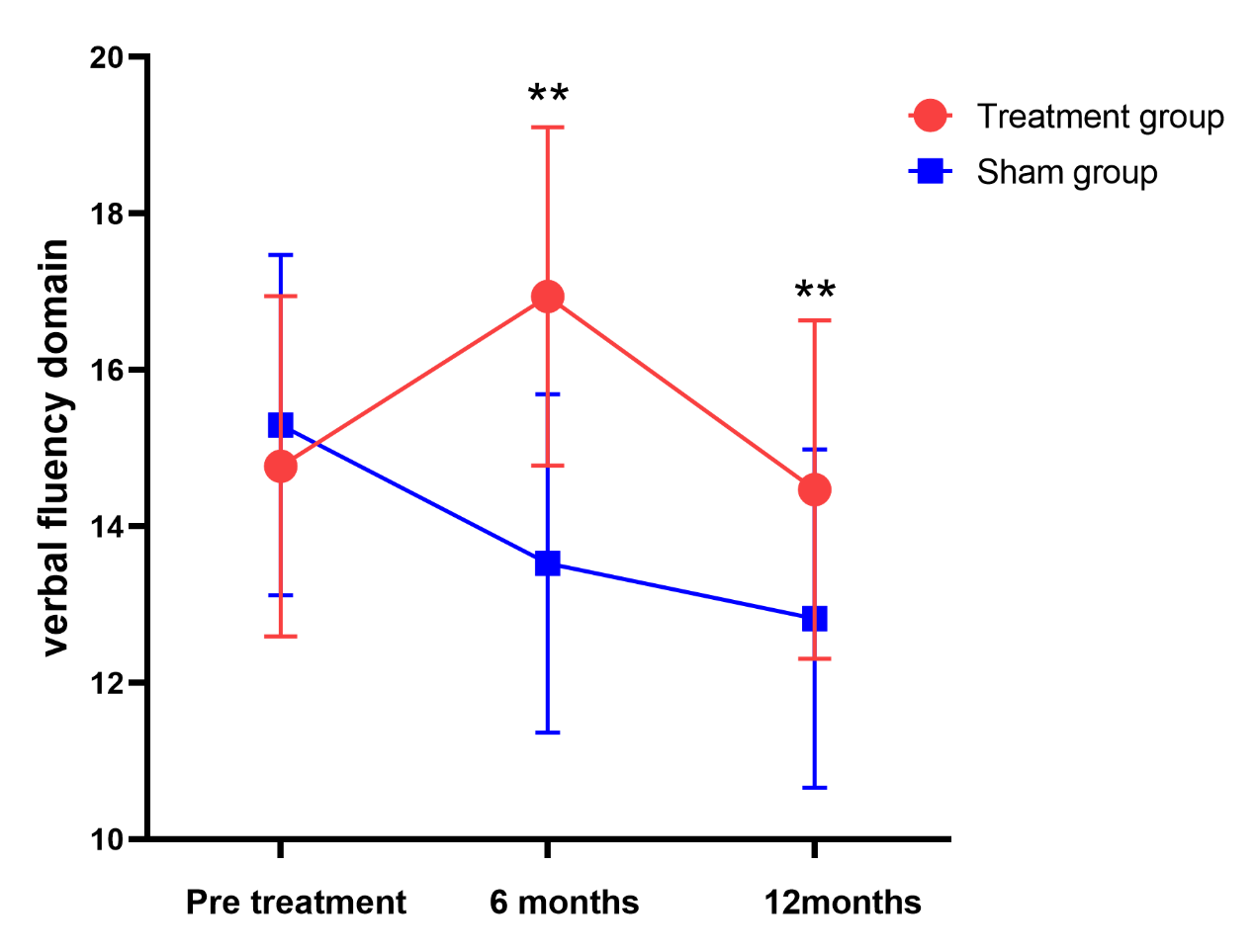


**Figure 10：Changes in the ECAS verval fluency domain in the Treatment group and Sham group**

**Data are presented as estimated mean and standard error from a linear mixed model. *P**<**0.05, **P**<**0.01.**


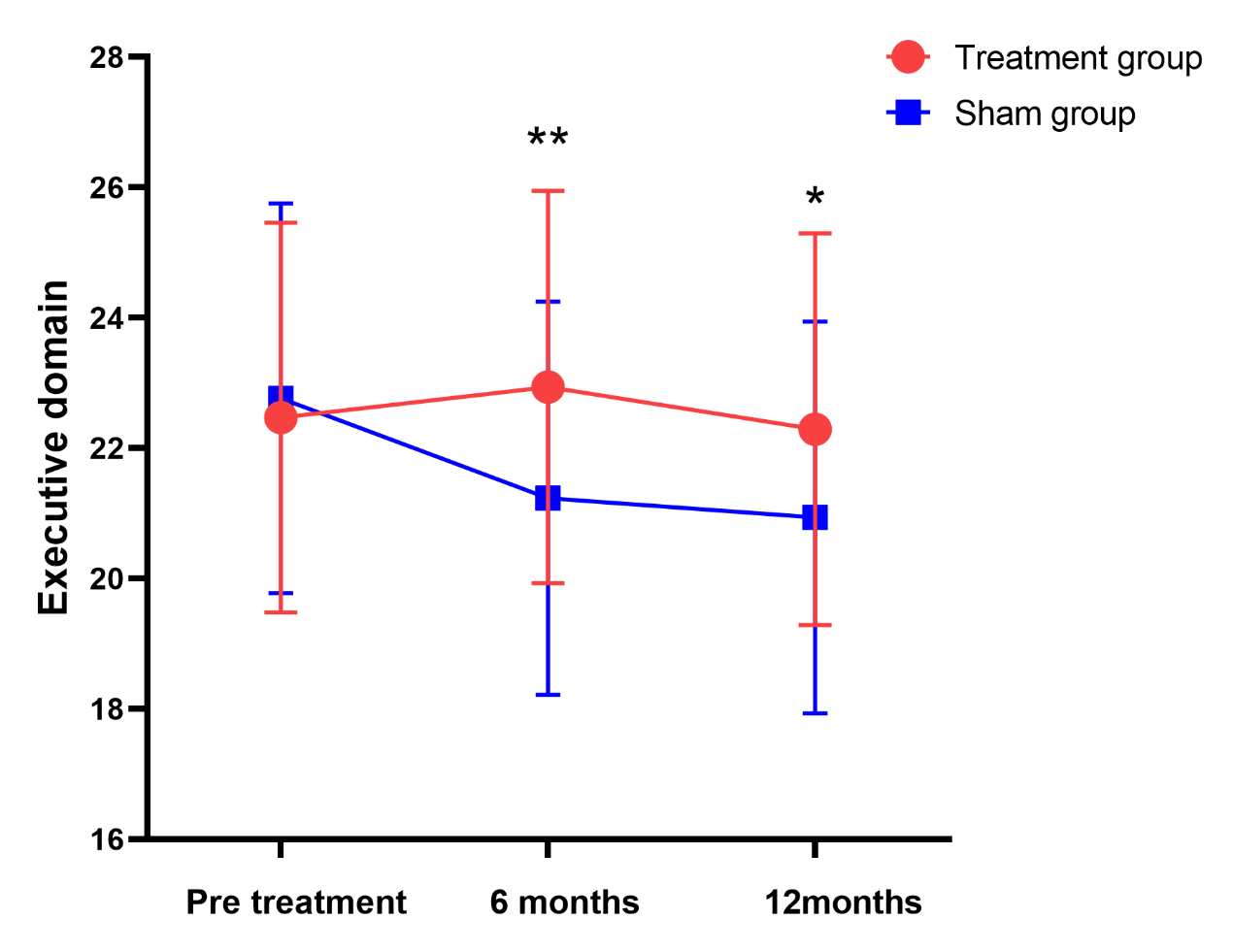


**Figure 11：Changes in the ECAS executive domain in the Treatment group and Sham group**

**Data are presented as estimated mean and standard error from a linear mixed model. *P**<**0.05, **P**<**0.01.**


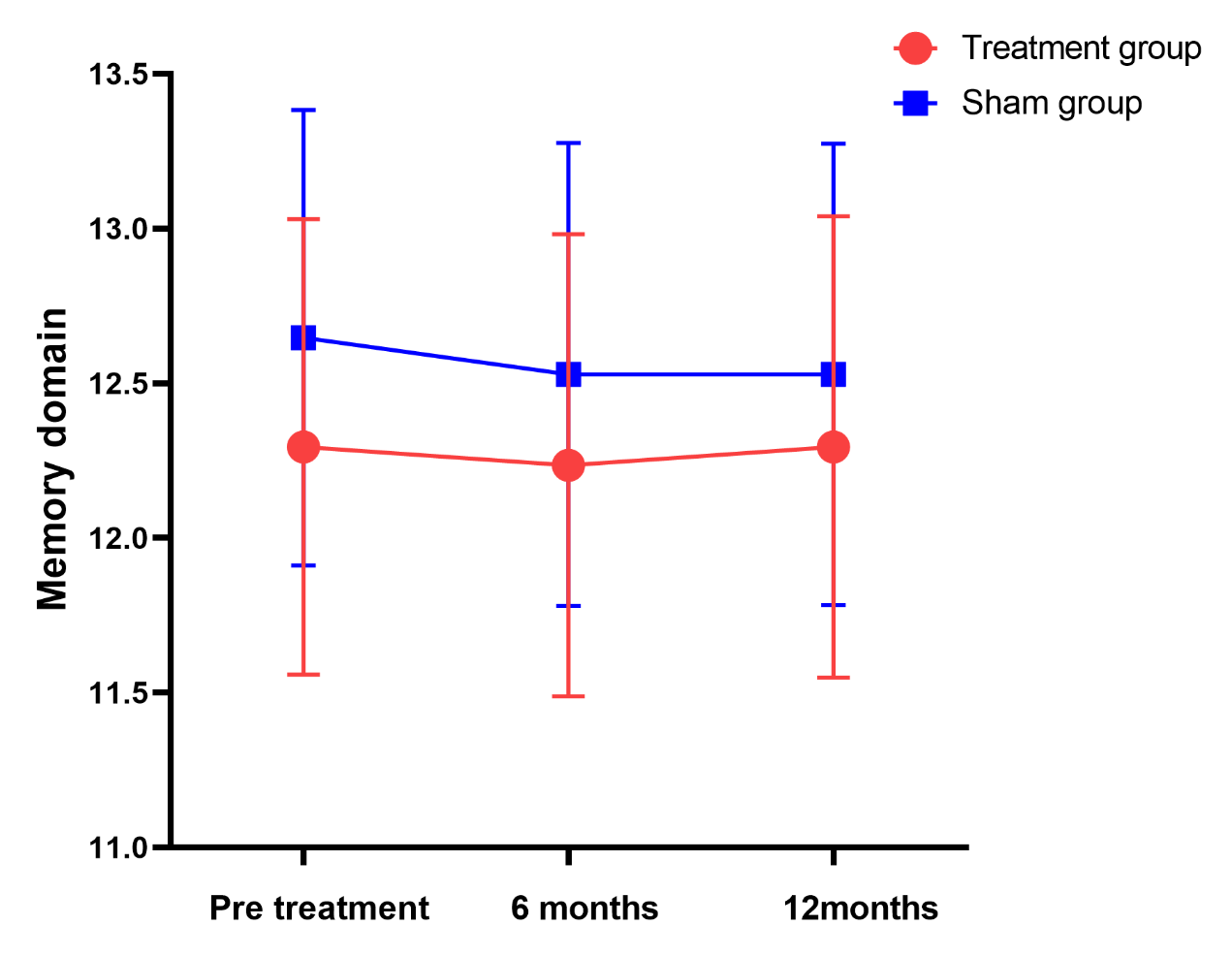


**Figure 12：Changes in the ECAS memeory domain in the Treatment group and Sham group**

**Data are presented as estimated mean and standard error from a linear mixed model. *P**<**0.05, **P**<**0.01.**


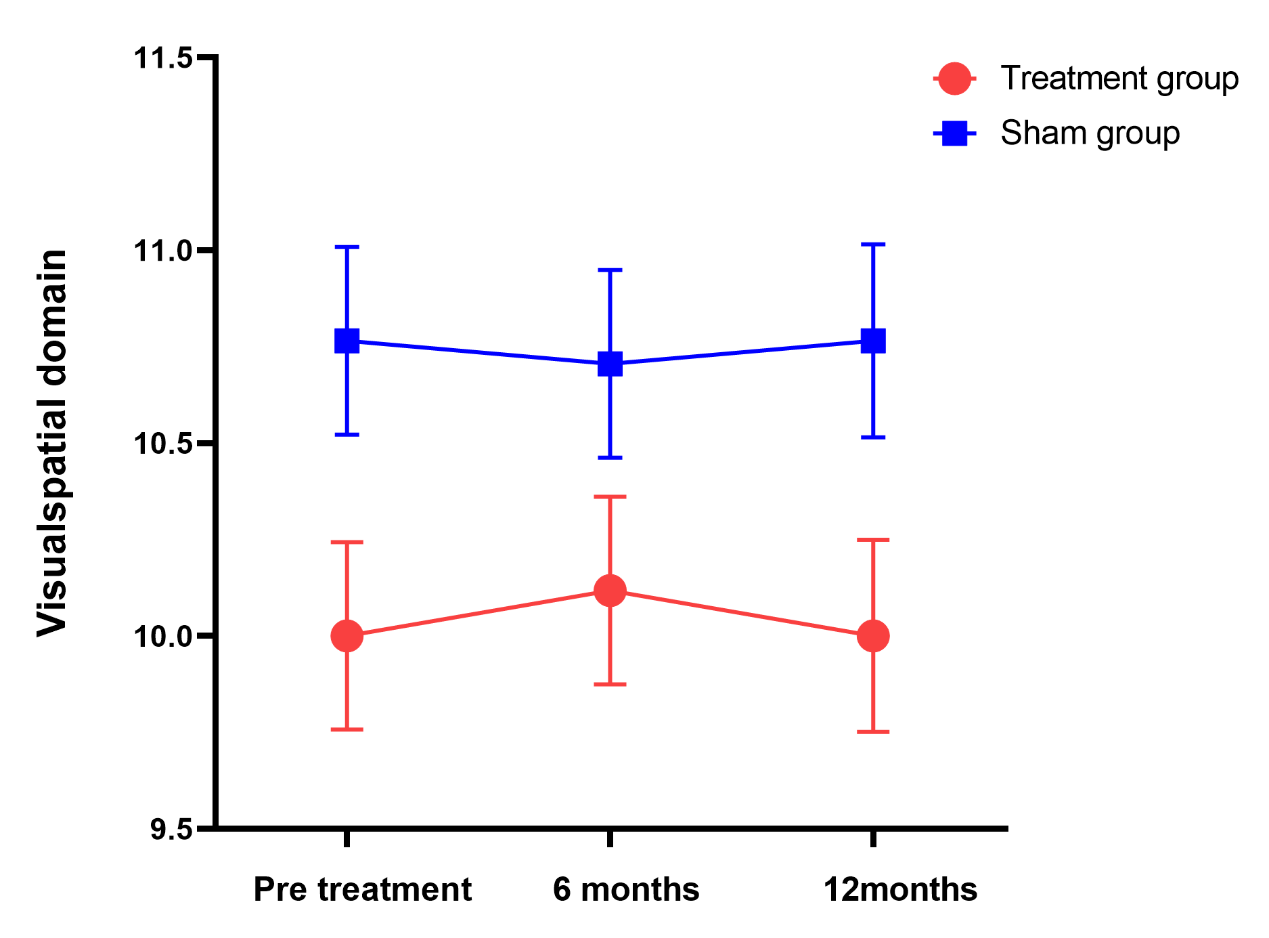


**Figure 13：Changes in the ECAS visuaspatial domain in the Treatment group and Sham group**

**Data are presented as estimated mean and standard error from a linear mixed model. *P**<**0.05, **P**<**0.01.**

**Table 5：Data for the changes in the ECAS language domain in the treatment group and sham group**

|  | Time points | Domain | Estimated mean | Standard error | Upper 95%  CI | Lower 95% CI | P值 |
| --- | --- | --- | --- | --- | --- | --- | --- |
| Sham treatment | Baseline | Language | 18.176 | 1.569 | 14.980 | 21.373 | N/A |
|  |  | Verbal fluency | 15.294 | 4.653 | 2.472 | 28.116 | N/A |
|  |  | Executive | 22.765 | 6.386 | -9.788 | 55.317 | N/A |
|  |  | Memory | 12.647 | 1.574 | 5.567 | 19.727 | N/A |
|  |  | Visualspatial | 10.765 | .520 | 9.706 | 11.824 | N/A |
| Sham treatment | 6 month | Language | 18.118 | 1.585 | 14.889 | 21.346 | N/A |
|  |  | Verbal fluency | 13.529 | 4.620 | .751 | 26.308 | N/A |
|  |  | Executive | 21.235 | 6.441 | -9.044 | 51.515 | N/A |
|  |  | Memory | 12.529 | 1.597 | 6.073 | 18.986 | N/A |
|  |  | Visualspatial | 10.765 | .520 | 9.706 | 11.824 | N/A |
| Sham treatment | 12 month | Language | 18.059 | 1.592 | 14.816 | 21.302 | N/A |
|  |  | Verbal fluency | 12.824 | 4.625 | .852 | 24.795 | N/A |
|  |  | Executive | 20.941 | 6.425 | -1.073 | 42.955 | N/A |
|  |  | Memory | 12.529 | 1.593 | 5.102 | 19.957 | N/A |
|  |  | Visualspatial | 10.706 | .534 | 9.618 | 11.794 | N/A |
| rTMS treatment | Baseline | Language | 19.941 | 1.569 | 16.745 | 23.137 | N/A |
|  |  | Verbal fluency | 14.765 | 4.653 | 1.943 | 27.587 | N/A |
|  |  | Executive | 22.471 | 6.386 | -10.082 | 55.023 | N/A |
|  |  | Memory | 12.294 | 1.574 | 5.214 | 19.374 | N/A |
|  |  | Visualspatial | 10.000 | .520 | 8.941 | 11.059 | N/A |
| rTMS treatment | 6 month | Language | 19.941 | 1.585 | 16.713 | 23.170 | 0.325 |
|  |  | Verbal fluency | 16.941 | 4.620 | 4.163 | 29.719 | 0.001** |
|  |  | Executive | 22.941 | 6.441 | -7.338 | 53.221 | 0.005** |
|  |  | Memory | 12.235 | 1.597 | 5.779 | 18.692 | 0.559 |
|  |  | Visualspatial | 10.000 | .520 | 8.941 | 11.059 | 1.000 |
| rTMS treatment | 12 month | Language | 19.882 | 1.592 | 16.640 | 23.125 | 0.559 |
|  |  | Verbal fluency | 14.471 | 4.625 | 2.499 | 26.442 | 0.001** |
|  |  | Executive | 22.294 | 6.425 | .280 | 44.308 | 0.016* |
|  |  | Memory | 12.294 | 1.593 | 4.866 | 19.722 | 0.154 |
|  |  | Visualspatial | 10.118 | .534 | 9.029 | 11.206 | 0.086 |


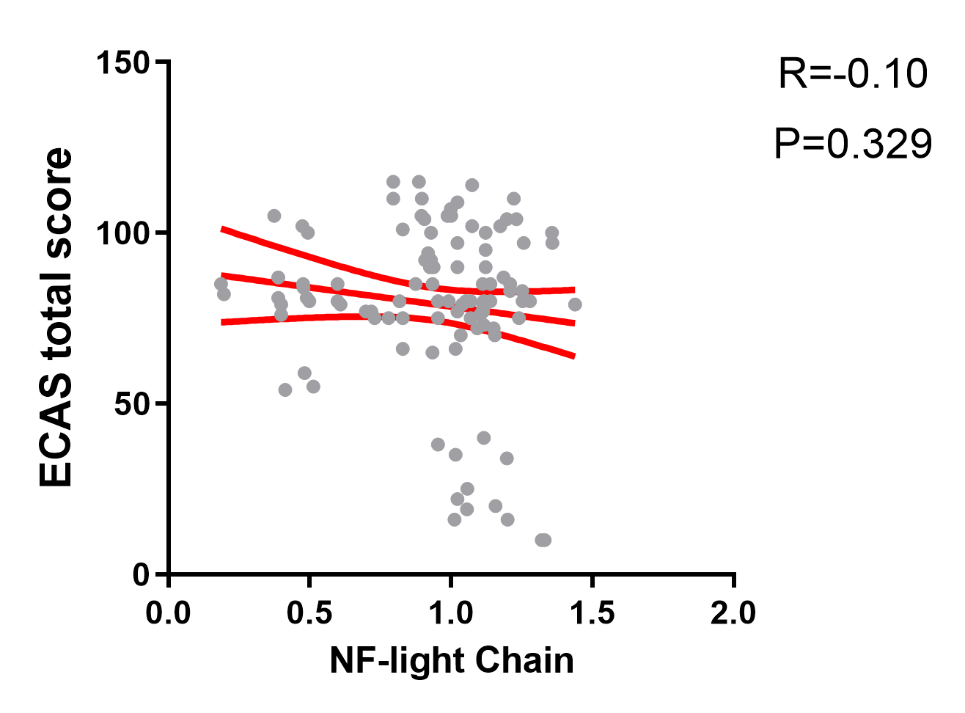


**Figure 14: NF-L correlation with ECAS score**

**No significant correlation was found between the level of plasma NF-L and ECAS scores (P**=**0.329). The solid red line and red curve line are the simple linear regression and its 95% confidence interval, respectively.**

**Table 6：Benjamini-Hochberg Adjusted P values for the multiple correlation analysis**

| Variable | Variable | Original P value for correlation analysis | Benjamini-Hochberg Adjusted P value | Significant using an FDR of 0.05? |
| --- | --- | --- | --- | --- |
| ECAS | ALSFRS-R | 0.008 | 0.032 | Yes |
| ECAS | Zarit Score | 0.016 | 0.032 | Yes |
| Zarit score | ALSFRS-R | 0.025 | 0.033 | Yes |
| ECAS | NF-L | 0.329 | 0.329 | No |

**Table 7：Random number table for the clinical trial**

| **Patien`t number** | **group** |
| --- | --- |
| 1` | A |
| 2 | B |
| 3 | A |
| 4 | B |
| 5 | B |
| 6 | A |
| 7 | B |
| 8 | A |
| 9 | B |
| 10 | B |
| 11 | B |
| 12 | B |
| 13 | A |
| 14 | B |
| 15 | A |
| 16 | B |
| 17 | B |
| 18 | A |
| 19 | B |
| 20 | B |
| 21 | B |
| 22 | A |
| 23 | B |
| 24 | B |
| 25 | A |
| 26 | A |
| 27 | A |
| 28 | B |
| 29 | A |
| 30 | B |
| 31 | B |
| 32 | B |
| 33 | B |
| 34 | B |
| 35 | B |
| 36 | A |
| 37 | A |
| 38 | A |
| 39 | B |
| 40 | A |
| 41 | B |
| 42 | A |
| 43 | A |
| 44 | B |
| 45 | B |
| 46 | A |
| 47 | B |
| 48 | A |
| 49 | B |
| 50 | A |
| 51 | A |
| 52 | A |
| 53 | B |
| 54 | B |
| 55 | B |
| 56 | A |
| 57 | A |
| 58 | B |
| 59 | A |
| 60 | A |
| 61 | A |
| 62 | B |
| 63 | B |
| 64 | A |
| 65 | A |
| 66 | B |
| 67 | A |
| 68 | A |
| 69 | A |
| 70 | A |
| 71 | A |
| 72 | B |
| 73 | B |
| 74 | B |
| 75 | A |
| 76 | A |
| 77 | A |
| 78 | B |
| 79 | A |
| 80 | A |

**A：treatment group B：sham group**
